# Cyclobutane-Containing Scaffolds as Useful Intermediates in the Stereoselective Synthesis of Suitable Candidates for Biomedical Purposes: Surfactants, Gelators and Metal Cation Ligands

**DOI:** 10.3390/ijms20184333

**Published:** 2019-09-04

**Authors:** Ona Illa, Albert Serra, Agustí Ardiaca, Xavier Herrero, Guillem Closa, Rosa M. Ortuño

**Affiliations:** Departament de Química, Universitat Autònoma de Barcelona, Cerdanyola del Vallès, 08193 Barcelona, Spain (A.S.) (A.A.) (X.H.) (G.C.)

**Keywords:** cyclobutane, amphiphiles, surfactants, gelators, cation ligands

## Abstract

Efficient and versatile synthetic methodologies are reported for the preparation of products that are suitable candidates to be used as surfactants, gelators for hydroxylic solvents or metal cation ligands, with potential use in several fields including biomedical applications. The common structural feature of all the synthesized products is the presence of a *cis* or *trans*-1,2- or *cis*-1,3-difunctionalized cyclobutane ring. In the two first cases, the key intermediates including enantiomerically pure 1,3-diamines and 1,3-amino alcohols have been prepared from β-amino acid derivatives obtained, in turn, from a chiral half-ester. This compound is also precursor of γ-amino esters. Furthermore, two kind of polydentate ligands have also been synthesized from a symmetric 1,5-diamine obtained from norpinic acid, which was easily prepared from commercial verbenone.

## 1. Introduction

Search for new molecules with suitable properties for biological purposes needs a rational synthetic design involving stereo- and chemoselective methodologies. Among target products, amphiphiles in general and, especially, surfactants and gelators play a key role in biomedicine. In addition, appropriate ligands to chelate cations are crucial for imaging technologies and radiopharmacy.

Amphiphiles are interesting and versatile compounds with usefulness in different fields. They can be classified in different families regarding their structure. Single head–single tail amphiphiles consist of an apolar tail and a polar head. Depending on the nature of the latter, they are classified as cationic, anionic or non-ionic amphiphiles [1]. They present a great potential for biomedical applications in targeted drug delivery systems (DDS) [1] and as non-viral vectors for gene therapy [2]. For the last purpose, cationic surfactants are especially interesting due to the fact of their properties that make them suitable to interact with DNA. Indeed, controlled DNA compaction and neutralization of their negatively charged phosphate groups is crucial to transfect into cells avoiding repulsive interactions with phospholipids in the cell membrane [3].

Otherwise, amphiphiles with properties as gelators and, in particular low-molecular-weight gelators (LMWG) are also valuable for the design and preparation of soft materials with utility in biochemical and biophysical studies [4] and applications in biomedicine, which include DDS, biosensors and tissue regeneration [5].

Imaging is nowadays a powerful tool in diagnosis and personalized medicine. In this area, contrast agents play a relevant role [6]. Many of them consist of appropriate metal complexes that must present specific requirements to assure their thermodynamic and kinetic stability to avoid metal leaching into the body. For this reason, the search for modifications in the structure of the ligands, trying to improve the properties and the effectiveness of the corresponding complexes, is the subject of a very active interdisciplinary research [7].

**Scheme 1:**
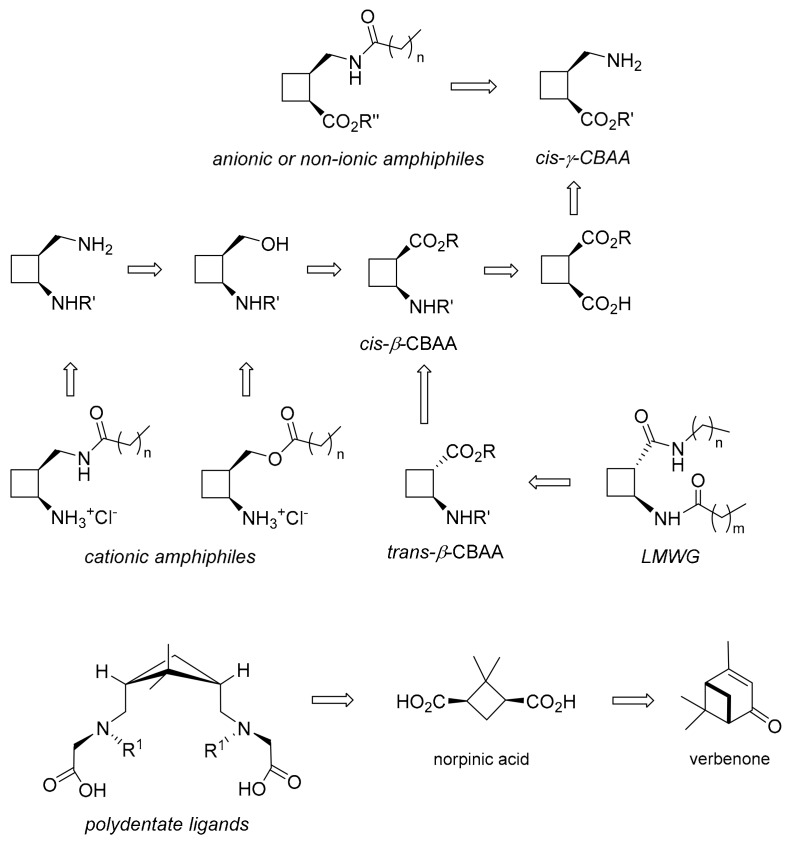
Retrosynthetic strategies to prepare the target products from suitable precursors.

Previously, we have developed efficient synthetic methodologies to prepare both 1,2- and 1,3- disubstituted cyclobutane derivatives, whose exploitation in the synthesis of several kinds of amphiphiles [8,9,10], organogelators producing chiral aggregates [11,12] and metal ligands [13] has been explored. The presence of the cyclobutane ring confers all these types of molecules with rigidity as well as with two stereogenic centers, the relative and absolute configurations of which can be controlled. Scheme 1 summarizes synthetic strategies to prepare some *cis*- and *trans*-1,2-cyclobutane derivatives from a chiral half-ester that is the common precursor to *cis*- [14] and *trans*-β-amino acids [15,16]. From these compounds, we synthesized peptides with properties as foldamers in solution [16,17,18] and/or LMWG [19,20] as well as neuropeptide Y (NPY) analogues [21]. Amino alcohols [22] and diamines [22,23] have also been prepared, some of them with application in organocatalysis [22,23]. The 1,3-diamine motif is found in natural products and also in the structure of ligands for metal catalysts. Despite the many synthetic methods described to prepare 1,2-diamine building blocks, the synthetic approaches to 1,3-diamines are more limited [24,25,26].

Concerning *cis*-1,3-disubstituted cyclobutane derivatives, we prepared organocatalysts [27] and other products as dendrimers [28] and cell penetrating peptides [29] starting from commercial verbenone, which provides polyfunctional chemical platforms that are highly useful scaffolds for synthetic applications.

In this article, we describe the utilization of *cis*- and *trans*-cyclobutane β-amino acids (β-CBAA) [30] in the stereoselective synthesis of novel chiral cationic surfactants and LMWG. Moreover, a new synthesis of *cis*-γ-CBAA derivatives is provided along with their application as synthetic precursors to anionic or non-ionic amphiphiles. Some examples are described herein to illustrate the usefulness of these methodologies (Scheme 1).

Moreover, highly rigid polydentate ligands for metal cations were synthesized as well from norpinic acid [31], which is easily obtained from verbenone (Scheme 1) or pinene. Related ligands, previously described in the literature, have formed gadolinium complexes with interesting properties as potential candidates to contrast agents for magnetic resonance imaging [32,33,34], or complexes with other cations of application in radiopharmacy [35] or for industrial purposes [36].

## 2. Results and Discussion

### 2.1. Amphiphiles from 1,2-Disubstituted Cyclobutane Scaffolds: Surfactants and LMWG

Scheme 1 shows two retrosynthetic strategies to prepare different families of single head–single tail amphiphiles. Cationic derivatives were synthesized from 1,3-diamines and 1,3-amino alcohols, respectively, as the immediate precursors. These compounds, in turn, were obtained from orthogonally protected *cis*-β-CBAA through selective transformations of the functional groups. A second synthetic route leads to the formation of *cis*-γ-CBAA as the key intermediates, which allows the preparation of anionic or non-ionic amphiphiles. Moreover, *trans*-β-CBAA, obtained through regioselective epimerization of the *cis* diastereomers, led to molecules which had structural features that conferred on them possible properties as organogelators (Scheme 1). Indeed, these compounds bear functional groups suitable for intermolecular hydrogen bonding and appropriate long alkyl-chains for van der Waals interactions. In addition, *trans* stereochemistry precludes the formation of intramolecular hydrogen bonds, thus favouring the interactions with solvents.

**Scheme 2:**
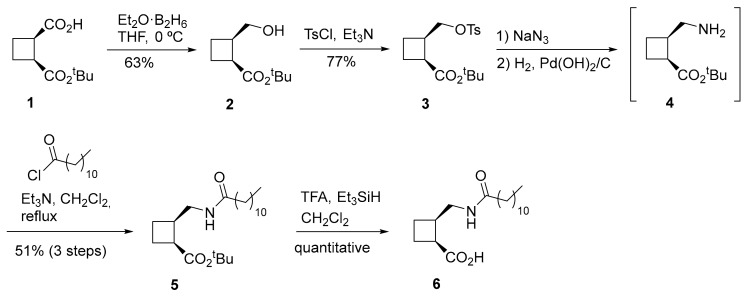
Synthetic route towards non-ionic or anionic amphiphiles.

The synthetic route towards non-ionic or anionic amphiphiles is depicted in Scheme 2. The carboxyl group in half-ester **1** [14] was selectively reduced with diborane in tetrahydrofuran, at 0 °C, providing primary alcohol **2** in 63% yield, which was activated towards nucleophilic displacement to introduce the amino group. Thus, treatment of **2** with tosyl chloride in the presence of triethylamine (TEA), giving tosylate **3** in 77% yield, and subsequent reaction with sodium azide followed by hydrogenation in the presence of 20% Pd(OH)_2_ on activated charcoal as a catalyst provided amino ester **4**. Reaction of this intermediate with lauryl chloride led to **5** in 51% yield (3 steps), which is representative of non-ionic amphiphiles. Moreover, deprotection of the carboxyl group, by acid cleavage of the *tert*-butyl ester with trifluoroacetic acid and triethylsilane in dichloromethane, afforded quantitatively **6**, which would be the direct precursor of an anionic amphiphile via deprotonation of the carboxylic acid.

**Scheme 3:**
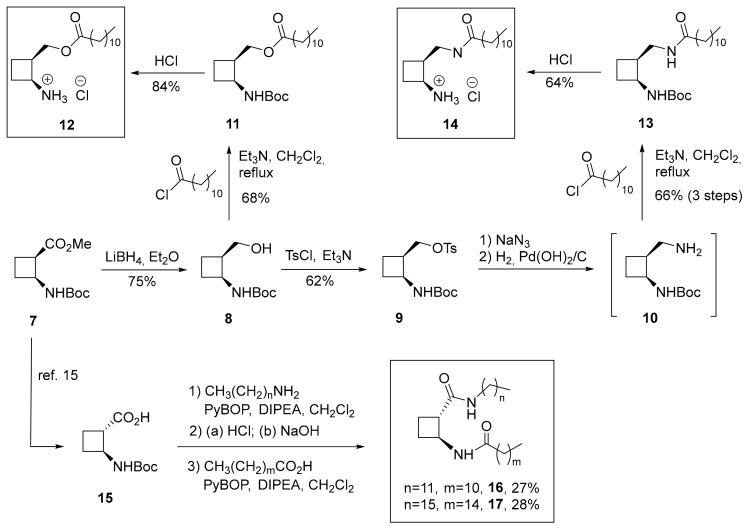
Synthesis of cationic amphiphiles and organogelators from *cis*- or *trans*-β-CBAA.

Scheme 3 shows the synthetic pathways to prepare cationic amphiphiles **12** and **14** and LMWG **18** and **19** from *cis*- and *trans*-CBAA, respectively. The methyl ester in precursor **7** [14] was reduced with lithium borohydride in ether to afford alcohol **8** in 75% yield. This compound was submitted to similar transformations as those described above, giving diamine **10**.

Separate reactions of amino alcohol **8** and diamine **10** with lauryl chloride and TEA under reflux of dichloromethane led to compounds **11** and **13**, respectively, in 66–68% yield. Treatment of these products with 2 N HCl in dichloromethane allowed to obtain cationic surfactants **12** and **14** in 84% and 64%, respectively.

The synthetic route for the preparation of LMWG **16** and **17** (Scheme 3) starts with coupling of previously described *trans*-β-CBAA **15 [15]** with dodecyl- or hexadecylamine, using (benzotriazol-1-yloxy)tripyrrolidinophosphonium hexafluorophosphate (PyBOP) as a coupling agent in the presence of diisopropylethylamine (DIPEA) in dichloromethane, at room temperature, afforded the corresponding amides that, in separate processes, were submitted to amine deprotection by treatment with HCl followed of aqueous NaOH to provide the free amines. Further coupling with lauric or palmitic acid, under similar conditions as before, led to diamides **16** and **17**, respectively, in 27–28% yield from **15**. Preliminary experiments showed that these compounds are good gelators for alcohols (MeOH, EtOH, i-PrOH) with minimum gelation concentration (*mgc*) values 8–47 mg/mL and biocompatible EtOH–water mixtures (up to 30% water) with *mgc* = 10–16 mg/mL. These data differentiate these LMWG from alkyl bisamides prepared from cyclobutane-1,2-dicarboxylic acid, which were insoluble in alcohols [11], and confer these products with very interesting features for future applications.

### 2.2. Highly Rigid Polydentate Ligands From 1,3-disubstituted Cyclobutane Scaffolds

**Scheme 4:**
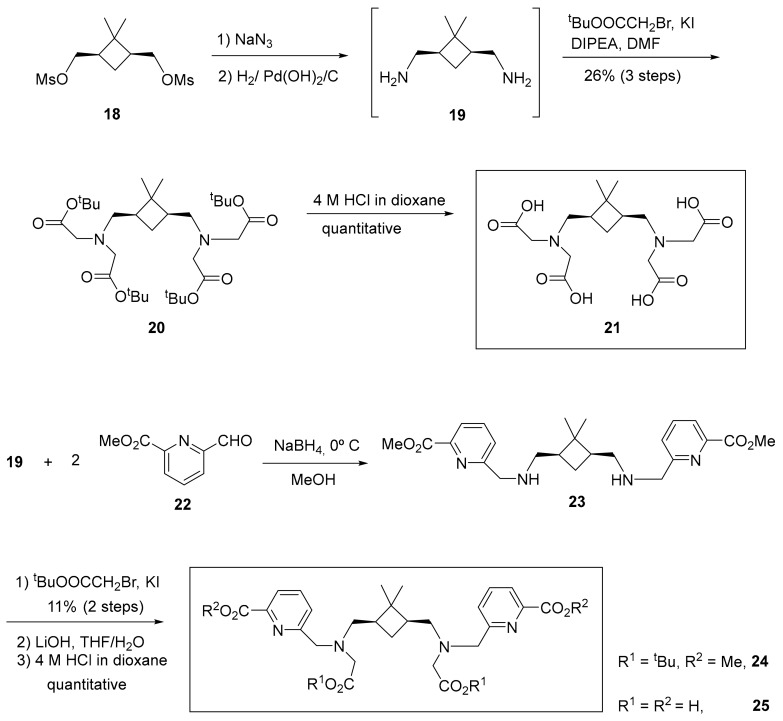
Synthesis of ligands from 1,3-disubstituted cyclobutane scaffolds.

1,5-Diamine **19** was synthesized from previously known dimesylate **18** (Scheme 4), which had been obtained from norpinic acid [31]. The procedure was similar to that described above for the preparation of amines **4** and **10**.

Tetraalkylation of diamine **19** with *tert*-butyl bromoacetate, in the presence of KI and DIPEA, using DMF as a solvent, afforded tetraester **20** in 26% yield. Tetraacid **21** was quantitatively obtained after treatment of **20** with 4 M HCl in dioxane.

Otherwise, cyclic or open-chain ligands bearing picolinate moieties have shown enhanced ability to coordinate metal cations making them useful for manifold applications including highly stable and kinetically inert contrast agents [33,34]. For this reason, we decided to synthesize ligand **25** according to Scheme 4.

Methyl 6-formylpicolinate, **22** [37], was submitted to reductive amination by treatment with 0.5 equivalents of diamine **19** and subsequent in situ reduction of the resultant double Schiff-base with sodium borohydride in methanol at 0 °C. Subsequent dialkylation was carried out as described before for the preparation of **20**, giving the analogue **24** in 11% yield for the two steps. Finally, saponification of the two methyl esters with 1.2 equivalents of LiOH in a 1:1 THF/water mixture at room temperature followed by treatment with 4 M HCl in dioxane, to remove the Boc protecting groups, led to ligand **25** in quantitative yield.

## 3. Materials and Methods

### 3.1. General Procedures

Melting points were determined using a Kofler apparatus model Reicher Austria^TM^ and are uncorrected. Specific rotations were recorded on a JASCO-715 optical polarimeter. Infrared spectra were recorded on a Bruker Tensor 27 spectrophotometer with sapphire-ATR (Golden Gate). Nuclear magnetic resonance spectra were recorded on an AC 250, AVANCE 360, or ARX Bruker apparatus. Mass spectra (MS) were obtained on a Bruker Squire 3000 micrOTOF spectrometer using ESI-MS (QTOF). Thin-layer chromatography was performed using ALUGRAM^®^ SIL G/UV_254_ aluminium sheets pre-coated with silica gel 60 (0.20 mm thickness) containing a fluorescent indicator at 254 nm (Macherey-Nagel, Düren, Germany). The TLC spots were visualized with a UV lamp or by staining with vanillin in 96% EtOH or with an acid KMnO_4_ solution. Column chromatography was carried out using PanReac^®^ silica gel (230–400 mesh) (Castellar del Vallès, Spain). Nitrogen was used as driving gas.

### 3.2. Experimental Section

#### 3.2.1. Synthesis of *tert*-Butyl (1*S*,2*R*)-2-Hydroxymethylcyclobutane-1-Carboxylate (**2**)

1 M B_2_H_6_ in THF (10.5 mL, 10.5 mmol) was added to a solution of half-ester **1** [14] (1.4 g, 6.9 mmoL) in anhydrous THF (70 mL) and the resultant mixture was stirred at 0 °C for 2 h under nitrogen atmosphere and 2 h at room temperature. The reaction was quenched with aqueous saturated NH_4_Cl (50 mL), then water (50 mL) was added and the mixture was extracted with EtOAc (4 × 80 mL). The combined organic layers were dried over MgSO_4_ and solvent was removed at reduced pressure. The residue was purified by flash chromatography (2:1 hexane/EtOAc) to afford alcohol **2** (0.85 g, 63% yield) as an oil. Rf = 0.27 (2:1 hexane/ethyl acetate); [α]_D_ −21.7 (c = 1.01 in CH_2_Cl_2_); ^1^H NMR (250 MHz, CDCl_3_) δ = 1.42 (s, 9H, O^t^Bu); 1.68 (m, 1H, H3); 1.99 (m, 2H, H3, H4); 2.23 (m, 1H, H3); 2.73 (m, 1H, H2); 3.02 (m, 1H, OH); 3.16 (m, 1H, H1); 3.58 (m, 1H, H5); 3.71 (m, 1H, H5) ppm; ^13^C NMR (62.5 MHz, CDCl_3_) δ = 21.3 (C3); 22.1 (C4); 28.4 (CBoc); 40.1 (C2); 41.2 (C1); 64.1 (C5); 81.3 (C7); 174.6 (C6) ppm; IR ν= 3411, 1720, 1152 cm^−1^; HRMS (ESI^+^) calcd m/z for C_10_H_18_O_3_ [M + Na]^+^: 209.1148, found: 209.1152 (Supplementary Materials).

#### 3.2.2. Synthesis of *tert*-Butyl (1*R*,2*S*)-2-Tosyloxymethylcyclobutane-1-Carboxylate (**3**)

A solution containing alcohol **2** (0.62 g, 3.3 mmol), *p*-toluenesulfonyl chloride (0.85 g, 4.3 mmol), 4-DMAP (0.1 g, 0.8 mmoL) and TEA (0.7 mL, 5.0 mmoL) in dry CH_2_Cl_2_ (30 mL) was stirred at room temperature overnight under nitrogen atmosphere. Then water (100 mL) was added and the mixture was extracted with CH_2_Cl_2_ (3 × 60 mL). Phases were separated and the organic layer was dried over MgSO_4_. Solvent was removed and the residue was purified by flash column chromatography (3:1 hexane/EtOAc) to afford tosylate **3** (0.87 g, 77% yield) as an oil. Rf = 0.30 (3:1 hexane/EtOAc); [α]_D_ +223.6 (c = 1.03 in CH_2_Cl_2_); ^1^H NMR (360 MHz, CDCl_3_) δ = 1.41 (s, 9H, ^t^Bu); 1.72 (m, 1H, H3); 2.02 (m, 2H, H4, H3); 2.23 (m, 1H, H3); 2.44 (s, 3H, H7′); 2.88 (m, 1H, H1); 3.16 (m, 1H, H2); 4.13 (dd, 1H, H5, *J* = 10.5 Hz, *J*’ = 7.2 Hz); 4.21 (dd, 1H, H5, *J* = 10.5 Hz, *J*’ = 7.2 Hz); 7.34 (d, *J* = 11.7 Hz, 2H, H3′, H5′); 7.75 (d, *J* = 11.7 Hz, 2H, H2′, H6′); ^13^C NMR (90 MHz, CDCl_3_) δ = 21.0, 21.3, 21.4 (C4, C7′, C3); 27.7 (CBoc); 35.6 (C1); 39.8 (C2); 70.5 (C5); 80.7 (C7); 127.6 (C2′, C6′); 129.5 (C3′, C5′); 132.8 (C4′); 144.4 (C1′); 172.1 (C6); IR ν = 2979.3, 1721.17, 1600.6 cm^−1^; HRMS (ESI^+^) calcd *m*/*z* for C_17_H_25_NO_5_S [M + Na]^+^: 363.1237, found: 363.1239.

#### 3.2.3. Synthesis of *tert*-Butyl (1*S*,2*R*)-2-Dodecanamidomethylcyclobutane-1-Carboxylate (**5**) through amine **4**

A mixture of tosylate **3** (0.84 g, 2.4 mmol) and sodium azide (0.48 g, 7.4 mmol) in anhydrous DMF (30 mL) was stirred and heated at 75 °C for 3 h under nitrogen atmosphere. Then EtOAc (60 mL) was added and the resultant solution was washed with water (5 × 50 mL). The combined aqueous layers were extracted with EtOAc (100 mL) and extract was washed with water (5 × 50 mL). The combined organic layers were dried over MgSO_4_ and concentrated (caution: azides can be explosive, so their solutions must be never evaporated to dryness). The residue was purified by column chromatography (6:1 hexane/ethyl acetate) to afford yellowish oil (Rf = 0.44, 6:1 hexane/ethyl acetate). The obtained oil in THF (20 mL) was hydrogenated under 1.9 atmospheres pressure in the presence of 20% Pd(OH)_2_/C as a catalyst (0.10 g). After 5 h the mixture was filtered over celite^®^ (Sigma-Aldrich, Darmstadt, Germany), which was washed with methanol (30 mL). Solvents were removed to afford a solid corresponding to partially protected diamine 4 that was used in the next step without further purification due to its instability.

A light protected solution of crude diamine **4** (0.10 g), dodecanoyl chloride (0.12 mL, 1.5 mmoL) and TEA (0.16 mL, 1.9 mmoL) in dry CH_2_Cl_2_ (10 mL) was stirred overnight under nitrogen atmosphere. The reaction mixture was successively washed with aqueous saturated NaHCO_3_ and brine. The organic layer was dried over MgSO_4_ and solvent was evaporated to dryness. The residue was washed with pentane affording compound **5** (0.1 g, 51% yield for the 3 steps) as a solid. m.p. 55–57 °C (pentane); [α]_D_ +10.7 (*c*= 1.06 in CH_2_Cl_2_); ^1^H NMR (250 MHz, CDCl_3_) δ = 0.90 (m, 3H, H12′); 1.28 (m, 16H, H4′, H5′, H6′, H7′, H8′, H9′, H10′, H11′); 1.51 (s, 9H, ^t^Bu); 1.64 (m, 2H, H3′); 1.75 (m, 1H, H3); 2.07 (m, 2H, H3, H4); 2.18 (m, 3H, H2′, H4); 2.80 (m, 1H, H2); 3.19 (m, 2H, H1, H5); 3.56 (m, 1H, H5); 6.10 (broad s, 1H, N*H*–C=O) ppm; ^13^C NMR (62.5 MHz, CDCl_3_) δ = 14.6 (C12′); 21.7–32.9 (C3, C4, C2′, C3′, C4′, C5′, C6′, C7′, C8′, C9′, C11′); 28.7 (CBoc); 37.4, 38.2 (C2′, C2); 41.3, 41.8 (C1, C5); 81.3 (C7); 173.7 (C1′); 174.3 (C6) ppm; IR ν = 3328, 2916, 1715, 1644, 1153 cm^−1^; HRMS (ESI^+^) calcd *m/z* for C_22_H_41_NO_3_ [M + Na]^+^: 390.2979, found: 390.2981.

#### 3.2.4. Synthesis of (1*S*,2*R*)-2-Dodecanamidomethylcyclobutane-1-Carboxylic Acid (**6**)

A solution containing ester **5** (90 mg, 0.2 mmol), TFA (0.25 mL, 3.8 mmoL), and triethylsilane (0.10 mL, 0.6 mmol) in dry CH_2_Cl_2_ (5 mL) was stirred at room temperature overnight under nitrogen atmosphere. Most of solvent was removed and the residue was lyophilized to afford acid **6** (74 mg, quantitative yield) as a solid. m.p. 48–50 °C; [α]_D_ +19.0 (c = 1.10 in CH_2_Cl_2_); ^1^H NMR (250 MHz, CDCl_3_) δ = 0.90 (t, 3H, H12′, *J* = 6.8 Hz); 1.27 (m, 16H, H4′, H5′, H6′, H7′, H8′, H9′, H10′, H3′); 1.61 (m, 2H, H3′); 1.81 (m, 1H, H3); 2.12 (m, 2H, H3, H4); 2.21 (t., 2H, H2′, *J* = 6.7 Hz); 2.35 (m, 1H, H4); 2.90 (m, 1H, H2); 3.29 (m, 1H, H5); 3.46 (m, 2H, H5, H1); 6.36 (broad s, 1H, NH–C=O); 7.61 (broad s, 1H, HO–C=O) ppm; ^13^C NMR (62.5 MHz, CDCl_3_) δ = 14.5 (C12′); 21.2 (C3); 23.1 (C4); 26.2–32.3 (C3′, C4′, C5′, C6′, C7′, C8′, C9′, C10′, C11′); 37.0 (C2′); 38.0 (C2); 40.7 (C5); 41.7 (C1); 175.1 (C1′); 178.6 (C6) ppm; IR ν = 3309, 1740, 1702, 1174 cm^−1^.

#### 3.2.5. Synthesis of *tert*-Butyl (1*S*,2*R*)-2-Hydroxymethylcyclobutane-1-Carbamate (**8**)

A solution of 2 M LiBH_4_ in THF (2.6 mL, 5.2 mmol) was added to a solution of amino ester **7** (1 g, 4.4. mmol) in Et_2_O (100 mL). The mixture was stirred at 0 °C for 3 h under nitrogen atmosphere. Aqueous saturated NH_4_Cl was added (60 mL) and the resultant mixture was stirred for 30 min, then water (50 mL) was added and the mixture was consecutively extracted with EtOAc (3 × 80 mL); the organic layer was dried over MgSO_4_ and evaporated to dryness. The residue was chromatographed on silica gel (3:1 hexane/EtOAc) to afford alcohol **8** (0.67 g, 75%) as a solid (Rf = 0.24, 3:1 hexane/EtOAc); crystals, m.p. 79–81 °C (hexane/EtOAc); [α]_D_ −77.8 (c = 1.02 in CH_2_Cl_2_); ^1^H NMR (250 MHz, CDCl_3_) δ = 1.46 (s, 9H, ^t^Bu); 1.63 (m, 1H, H3); 1.88 (m, 2H, H4, H3); 2.36 (m, 1H, H4); 2.71 (m, 1H, H2); 3.63 (dd, 1H, H5, *J* = 11.3 Hz, *J*’ = 4.3 Hz); 3.78 (dd, 1H, H5, *J* = 11.3 Hz, *J*’ = 4.3 Hz); 3.75 (m, 1H); 4.21 (m, 1H, H1); 5.12 (broad s, 1H, NHBoc) ppm; ^13^C NMR (62.5 MHz, CDCl_3_) δ = 19.2 (C3); 28.8 (C4); 28.7 (CBoc); 41.9 (C2); 48.1 (C1); 62.8 (C5); 80.1 (C7); 156.9 (C6) ppm; IR ν = 3311, 2976, 1691, 1174 cm^−1^; HRMS (ESI^+^) calcd m/z for C_10_H_19_NO_3_ [M + Na]^+^: 224.1257, found: 224.1260.

#### 3.2.6. Synthesis of (1*S*,2*R*)-1-*tert*-Butyloxycarbonylaminocyclobutane-1-Methyl Dodecanoate (**11**)

A solution of alcohol **8** (0.3 g, 1.5 mmoL), lauryl chloride (0.36 mL, 1.5 mmoL), and TEA (0.27 mL, 1.9 mmoL) in dry CH_2_Cl_2_ (15 mL) was heated to reflux overnight under nitrogen atmosphere. The reaction mixture was cooled to room temperature, washed once with aqueous saturated NaHCO_3_ (10 mL) and twice with brine, and dried over MgSO_4_. Solvent was removed and the residue was purified by flash chromatography (CH_2_Cl_2_) to give pure **11** (0.4 g, 68%) as a solid. m.p. 44–46 °C (CH_2_Cl_2_); [α]_D_ −63.9 (c = 0.97 in CH_2_Cl_2_); ^1^H NMR (360 MHz, CDCl_3_) δ = 0.87 (t, 3H, H12′, *J* = 8.4 Hz); 1.25 (m, 16H, H4′, H5′, H6′, H7′, H8′, H9′, H10′, H11′); 1.43 (s, 9H, tBu); 1.63 (m, 3H, H4, H3′); 1.92 (m, 2H, H3, H4); 2.33 (m; 3H, H2′, H3); 2.81 (m, 1H, H1); 4.16 (dd, 1H, H5, *J* = 13.8 Hz, *J*’ = 7.2 Hz); 4.31 (dd, 1H, H5, *J* = 13.8 Hz, *J*’ = 7.2 Hz); 4.30 (m, 1H, H2); 4.79 (broad s, 1H, NHBoc) ppm; ^13^C NMR (90 MHz, CDCl_3_) δ = 13.8 (C12′); 17.7 (C4); 22.2–31.6 (C3′, C4′, C5′, C6′, C7′, C8′, C9′, C10′, C11′); 26.6 (C4); 28.1 (CBoc); 34.1 (C2′); 38,6 (C1); 45.8 (C2); 63.4 (C5); 79.0 (C7); 154.7 (C6); 173.7 (C1′) ppm; IR ν = 3342, 2919, 1679, 1166 cm^−1^; HRMS (ESI^+^) calcd m/z for C_22_H_41_NO_4_ [M + Na]^+^: 406.2928, found: 406.2930.

#### 3.2.7. Synthesis of (1*S*,2*R*)-2-Dodecanoyloxymethylcyclobutane-1-Ammonium Chloride (**12**)

A solution of compound **11** (0.2 g, 0.5 mmol) and 2 N HCl in Et_2_O (1.56 mL, 3.1 mmol) in dry CH_2_Cl_2_ (15 mL) was stirred at room temperature for 24 h under nitrogen atmosphere. Most solvent was removed, and the residue was lyophilized to afford quantitatively a solid that was crystallized in pentane to provide surfactant **12** (0.14 g, 84% yield) as crystals. m.p. 86–88 °C (pentane); [α]_D_ +9.6 (c = 1.01 in MeOH); ^1^H NMR (360 MHz, CD_3_OD) δ = 0.91 (m, 3H, H12′); 1.30 (m, 16H, H4′, H5′, H6′, H7′, H8′, H9′, H10′, H11′); 1.63 (m, 2H, H3′); 1.81 (m, 1H, H3); 2.17 (m, 2H, H4, H3); 2.39 (m, 3H, H2′, H4); 2.95 (m, 1H, H2); 3.95 (m, 1H, H1); 4.32 (m, 2H, H5); ^13^C NMR (90 MHz, CD_3_OD) δ = 13.1 (C12′); 17.9 (C3); 22.3–29.3 (C3′, C4′, C5′, C6′, C7′, C8′, C9′, C10′, C11′); 31.7 (C4); 33.4 (C2′); 36.4 (C2); 46.6 (C5); 62.3 (C1); 173.8 (C1′) ppm; IR ν = 3370, 2919, 1740, 1112 cm^−1^; HRMS (ESI^+^) calcd *m*/*z* for C_17_H_34_ClNO_2_ [M]^+^: 319.2348, found: 319.2346.

#### 3.2.8. Synthesis of *tert*-Butyl (1*S*,2*R*)-2-Tosyloxymethylcyclobutane-1-Carboxylate (**9**)

Following a procedure similar to that described above for the preparation of tosylate **3**, compound **9** (0.57 g, 62% yield) was obtained as a solid. Rf = 0.28 (6:1 hexane/EtOAc); crystals, m.p. 87–88 °C (ethyl acetate/hexane); [α]_D_ −102.9 (c = 1.05 in CH_2_Cl_2_); ^1^H NMR (360 MHz, CDCl_3_) δ = 1.41 (s, 9H, ^t^Bu); 1.58 (m, 1H, H3); 1.88 (m, 2H, H4, H3); 2.27 (m, 1H, H3); 2.45 (s, 3H, H7′); 2.75 (m, 1H, H1); 4.17 (m, 2H, H5); 4.33 (m, 1H, H2); 4.77 (broad s, 1H, NHBoc); 7.34 (d, *J* = 11.7 Hz, 2H, H3′, H5′); 7.75 (d, *J* = 11.7 Hz, 2H, H2′, H6′) ppm; ^13^C NMR (90 MHz, CDCl_3_) δ = 17.6 (C4); 21.4 (C7′); 28.0 (CBoc); 28.1 (C3); 38.7 (C1); 45.8 (C2); 69.8 (C5); 79.1 (C7); 127.6 (C2′, C6′); 129.6 (C3′, C5′); 132.6 (C4′); 144.6 (C1′); 154.8 (C6) ppm; IR: ν = 3311, 2976, 1691, 1174 cm^−1^; HRMS (ESI^+^) calcd m/z for C_17_H_25_NO_5_S [M + Na]^+^: 378.1346, found: 378.1344.

#### 3.2.9. Synthesis of *tert*-Butyl (1*S*,2*S*)-2-Dodecanamidomethylcyclobutane-1-Carbamate (**13**) through diamine **10**

Following a similar procedure than that described above for the preparation of **5**, compound **13** (66% overall yield for the three steps) was obtained as crystals. m.p. 102-104 °C (pentane); [α]_D_ −40.2 (c = 1.01 in CH_2_Cl_2_); ^1^H NMR (360 MHz, CDCl_3_) δ = 0.89 (t, 3H, H12′, J=5.7 Hz); 1.25 (m, 16H, H4′, H5′, H6′, H7′, H8′, H9′, H10′, H11′); 1.44 (s, 9H, tBu); 1.61 (m, 3H, H3, H3′); 1.80 (m, 1H, H4); 1.92 (m, 1H, H3); 2.13 (t., 2H, H2′, *J* = 6.3 Hz); 2.36 (m, 1H, H4); 2.59 (m, 1H, H2); 3.15 (m, 1H, H5); 3.52 (m, 1H, H5); 4.17 (m, 1H, H1); 4.99 (broad s, 1H, NHBoc); 6.12 (broad s, 1H, NH-C=O) ppm; ^13^C NMR (90 MHz, CDCl_3_) δ = 14.1 (C12′); 20.1 (C3); 26.6 (C4); 22.7–29.6 (C2′, C3′, C4′, C5′, C6′, C7′, C8′, C9′, C11′); 28.4 (CBoc); 36.9 (C10′); 39.1–40.0 (C2, C5); 47.2 (C1); 79.7 (C7); 156.2 (C6); 173.3 (C1′) ppm; IR ν = 3342, 2919, 1679 cm^−1^; HRMS (ESI^+^) calcd *m*/*z* for C_22_H_42_N_2_O_3_ [M + Na]^+^: 405.3088, found: 405.3088.

#### 3.2.10. Synthesis of (1*S*,2*R*)-2-Dodecanamidomethylcyclobutane-1-Ammonium Chloride (**14**)

A solution of compound **13** (0.2 g, 0.5 mmol) and 2 N HCl in Et_2_O (1.56 mL, 3.1 mmol) in dry CH_2_Cl_2_ (15 mL) was stirred at room temperature for 24 h under nitrogen atmosphere. Most solvent was removed, and the residue was lyophilized to afford quantitatively a solid that was crystallized in pentane to provide amphiphile **14** (0.1 g, 64% yield). Crystals, m.p. 98–100 °C (pentane); [α]_D_ +8.7 (c = 1.05 in MeOH); ^1^H NMR (360 MHz, CD_3_OD) δ = 0.91 (m, 3H, H12′); 1.30 (m, 16H, H4′, H5′, H6′, H7′, H8′, H9′, H10′ H11′); 1.61 (m, 2H, H3′); 1.98 (m, 2H, H3, H4); 2.09 (m, 1H, H3); 2.23 (t, 2H, H2′, J=6.3 Hz); 2.39 (m, 1H, H4); 2.79 (m, 1H, H1); 3.16 (m, 1H, H5); 3.44 (m, 1H, H5); 3.75 (m, 1H, H1) ppm; ^13^C NMR (90 MHz, CD_3_OD) δ = 13.0 (C12′); 20.2 (C3); 22.3 (C4); 22.7–29.6 (C3′, C4′, C5′, C6′, C7′, C8′, C9′, C10′, C11′); 35.5 (C2′); 37.8–38.6 (C2, C5); 47.2 (C1); 176.2 (C1′) ppm; IR ν = 3342, 2918, 1679 cm^−1^; HRMS (ESI^+^) calcd m/z for C_17_H_35_N_2_O [M]^+^: 283.2744, found: 283.2745.

#### 3.2.11. Synthesis of Diamides **16** and **17**. General Procedure

To a solution of compound **15** [15] (0.18 g, 0.84 mmol) in anhydrous CH_2_Cl_2_ (27.0 mL), DIPEA (0.44 mL, 2.52 mmoL) and PyBOP (0.55 g, 1.06 mmoL) were added. After 15 min stirring at room temperature, dodecylamine or hexadecylamine (0.92 mmoL) dissolved in anhydrous CH_2_Cl_2_ (10.0 mL) was added and the resulting mixture was stirred at room temperature for 18 h. The solution was diluted with CH_2_Cl_2_ (10.0 mL) and washed with aqueous saturated NaHCO_3_ (30.0 mL). The organic layer was dried over MgSO_4_ and evaporated under vacuum. The residue was purified by column chromatography (1:1 hexane-EtOAc) to afford the corresponding pure amide. A mixture containing this compound (0.40 mmoL), 2 N HCl in Et_2_O (20 mmoL) and anhydrous CH_2_Cl_2_ (10.0 mL) was stirred at room temperature for 36 h. The solvent was evaporated at reduced pressure to afford the corresponding amine hydrochloride, which was dissolved in a 1:1 mixture of 0.25 M NaOH-CH_2_Cl_2_ and stirred vigorously for 15 min. Then the reaction mixture was extracted with CH_2_Cl_2_ (3 × 10 mL). The combined organic layers were dried over MgSO_4_ and solvent was removed to afford the respective free amine. This was coupled with lauryl or palmitic acid, respectively, as described above for the first coupling procedure. After purification by column chromatography (5% MeOH-CH_2_Cl_2_) the corresponding pure diamides were obtained as solid products.

##### (1*S*,2*S*)-*N*-Dodecyl-2-Dodecanamidocyclobutane-1-Carboxamide (**16**)

27% overall yield from **15**. Crystals, m.p. 74–79 °C (CH_2_Cl_2_); [α]_D_ +2.1 (c = 1.05 in CH_2_Cl_2_); ^1^H NMR (360 MHz, CDCl_3_) δ = 0.92 (t, *J* = 6.4 Hz, 6H, H19, H18′), 1.28 (s, 34H, H10-18, H10′-17′), 1.60 (m, 4H, H9, H9′), 1.81–2.00 (m, 2H, H4-3), 2.19 (m, 4H, H3-4, H8′), 2.90 (q, *J* = 9.1 Hz, 1H, H5), 3.24 (m, 2H, H8), 4.33 (m, 1H, H2), 5.88 (broad s, 1H, N1H), 8.36 (broad s, 1H, N7H) ppm; ^13^C NMR (90 MHz, CDCl_3_) δ = 13.8 (C19, C19′); 18.6 (C4); 22.3 (C18, C18′); 24.1 (C9-16,C10′-16′); 25.4 (C9-16, C10′-16′); 26.8 (C3); 29.0-29.4 (C9-16,C10′-16′); 31.7 (C17,C17′); 36.1 (C17, C17′); 39.1 (C8); 47.7 (C2); 49.7 (C5, C9′); 172.5 (C7); 174.1 (C8′) ppm; IR ν = 2952, 1685, 1638 cm^−1^; HRMS (ESI^+^) calcd *m*/*z* for C_29_H_56_N_2_O_2_ [M + H]^+^: 465.4415, found: 465.4397.

##### (1*S*,2*S*)-*N*-Hexadecyl-2-Palmitamidocyclobutane-1-Carboxamide (**17**)

28% overall yield from **15**. Crystals, m.p. 116-118 °C (CH_2_Cl_2_); [α]_D_ +6.0 (*c* = 1.04 in CH_2_Cl_2_); ^1^H NMR (360 MHz, CDCl_3_) δ = 0.88 (t, *J* = 5.5 Hz, 6H, H23, H22′), 1.26 (s, 50H, H10-22, H10′-21′), 1.61 (m, 4H, H9, H9′), 1.77–2.00 (m, 2H, H4-3), 2.18 (m, 4H, H3-4, H8′), 2.89 (q, *J* = 8.7 Hz, 1H, H5), 3.21 (m, 2H, H8), 4.32 (m, 1H, H2), 5.80 (d, *J* = 6.6 Hz, 1H, N1*H*), 8.34 (s, 1H, N7*H*) ppm; ^13^C NMR (90 MHz, CDCl_3_) δ = 13.9 (C23, C23′); 18.6 (C4); 22.2 (C22, C22′); 24.1 (C9-20, C10′-20′); 25.4 (C9-20, C10′-20′); 26.8 (C3); 29.0–29.4 (C9-20, C10′-20′); 31.6 (C21, C21′); 39.3 (C8); 47.4 (C2); 49.7 (C5, C9′); 172.2 (C7); 173.9 (C8′) ppm; IR ν = 3293, 2955, 2849, 1686, 1644 cm^−1^; HRMS (ESI^+^) calcd *m/z* for C_37_H_72_N_2_O_2_ [M + Na]^+^: 599.5486, found: 599.5478.

#### 3.2.12. Synthesis of Tetra-*tert*-Butyl *N,N,N’,N’*-[((1*S*,3*R*)-2,2-Dimethylcyclobutane-1,3-diyl) Bis(Methylene)Bis(Azanetriyl)] Tetraacetate (**20**)

Diamine **19** was prepared from dimesylate **18** [31] as described above for the synthesis of diamine **4** and used in the next step without further purification. *tert*-Butyl bromoacetate (3.7 mL, 25.1 mmoL) was added dropwise to a mixture of crude diamine **19** (0.45 g, 3.2 mmoL), potassium iodide (2.8 g, 17 mmoL), and DIPEA (8.7 mL, 50.2 mmoL) in anhydrous DMF (15 mL). The resultant solution was stirred at room temperature for 96 h under nitrogen atmosphere. The mixture was diluted with dichloromethane (150 mL) and washed with sat. K_2_CO_3_ (2 × 30 mL) and brine (40 mL). The organic phase was dried over MgSO_4_ and the solvent was evaporated to afford a yellow oil. The crude product was purified by column chromatography (4:1 Hexane/EtOAc) to obtain a colourless oil (0.48 g, 26% overall yield from dimesylate **18**). ^1^H NMR (250 MHz, CDCl_3_) δ = 0.92 (s, 3H, H6/5); 1.09 (s, 3H, H5/6); 1.47 (s, 36H, H11); 2.06 (m, 3H, H1, H3, H4); 2.66 (m, 4H, H7); 3.39 (s, 8H, H8); ppm; ^13^C NMR (62.5 MHz, CDCl_3_) δ = 16.0 (C6); 28.0 (C5); 29.6 (C11); 30.0 (C2); 40.0 (C4); 40.9 (C1, C3); 54.5 (C7); 55.9 (C8); 80.8 (C10); 170.7 (C9) ppm; IR ν = 1727, 1146 cm^−1^; HRMS (ESI^+^) calcd *m*/*z* for C_32_H_58_N_2_O_8_ [M + H]^+^: 599.4266, found: 599.4252.

#### 3.2.13. Synthesis of *N,N,N’,N’*-[((1*S*,3*R*)-2,2-dimethylcyclobutane-1,3-diyl) bis(methylene)-bis(azanetriyl)] tetraacetic acid (**21**)

A solution of tetraester **20** (0.20 g, 0.3 mmoL) in 4 M HCl in dioxane (12 mL, 48 mmoL) was stirred at room temperature for 20 h. Solvent was removed at reduced pressure, then water (2 mL) was added and the mixture was lyophilized to afford **21** as a solid (0.13 g, quantitative). ^1^H NMR (250 MHz, CDCl_3_) δ = 0.85 (s, 3H, H5/6); 1.03 (s, 3H, H5/6), 1.14–1.29 (m, 1H, H4); 1.94–2.11 (m, 3H, H1,3,4); 2.48 (m, 2H, H7); 2.62 (m, 2H, H7); 3.95 (s, 6H, H15); 3.77–4.06 (m, 4H, H8); 7.54 (m, 2H, H9); 7.77 (m, 2H, H10); 7.97 (m, 2H, H11) ppm; ^13^C NMR (62.5 MHz, CDCl_3_) δ = 15.1 (C6); 27.6 (C5); 29.5 (C4); 37.0 (C2); 41.2 (C3); 55.5 (C7); 56.9 (C8); 168.7 (C9) ppm; IR ν = 1730 cm^−1^; HRMS (ESI^+^) calcd *m*/*z* for C_16_H_26_N_2_O_8_ [M - H]^−^: 373.1616, found: 373.1626.

#### 3.2.14. Synthesis of Dimethyl 6,6′-[{2,2-Dimethylcyclobutane-1,3-diylbis[(2-*tert*-butoxy-2-oxoethyl)- azanemethylenediyl]}bis(methylene)] dipicolinate (**24**) through compound (**23**)

A solution of diamine **19** (0.6 g, 4.3 mmol) and methyl 6-formyl picolinate **22** [37] (0.14 g, 8.6 mmoL) in MeOH (20 mL) was stirred at room temperature for 5 h. Then, the solution was cooled with ice and NaBH_4_ (0.26 g, 7 mmoL) and MeOH (20 mL) were added. The resultant ice-cooled mixture was stirred for 2 h. Aqueous saturated NaHCO_3_ (30 mL) was added and most methanol was removed. The solution was extracted with CH_2_Cl_2_ (4 × 50 mL), the combined organic layers were dried over MgSO_4_ and solvent was removed to afford crude compound **23** that was identified by its ^1^H NMR spectrum and used in the next step without further purification. ^1^H NMR (250 MHz, CDCl_3_) δ = 0.91 (s, 3H, H4/5); 1.09 (s, 3H), 1.14–1.29 (m, 1H, H1); 1.94-2.11 (m, 3H, H1,2,3); 2.48 (m, 2H, H6/7); 2.62 (m, 2H, H6/7); 3.95 (s, 6H, H12); 3.77–4.06 (m, 4H, H8); 7.54 (d, *J* = 7.8 Hz, 2H, H9/11); 7.77 (t, *J* = 7.7 Hz, 2H, H10); 7.97 (d, *J* = 7.7 Hz, 2H, H9/11).

Using the same procedure as that described above to prepare **20**, crude **23** (0.5 g) was made to react with *tert*-butyl bromoacetate affording a reaction crude, which was purified by column chromatography (3:1 to 1:1 hexane/EtOAc) affording tetraester **24** (0.35 g, 11% yield for the two steps) as a yellowish oil. ^1^H NMR (400 MHz, CDCl_3_) δ = 0.63 (s, 3H, H5/6); 0.88 (s, 3H, H5/6); 1.13 (m, 1H, H4); 1.28 (s, 18H, H19); 1.88 (m, 3H, H1,3,4); 2.44 (m, 4H, H7); 3.07 (m, 4H, H8/16); 3.79 (m, 4H, H8/16); 3.82 (s, 6H, H15); 7.68 (m, 4H, H10,11); 7.84 (m, 2H, H12) ppm; ^13^C NMR (62.5 MHz, CDCl_3_) δ = 15.8 (C5,6); 27.9 (C19); 29.3 (C4); 29.9 (C5,6); 39.6 (C3/11); 40.3 (C2); 40.5 (C1,3); 52.5 (C7); 54.8 (C15); 56.1 (C16); 60.2 (C8); 80.6 (C18); 123.2 (C10); 125.7 (C12); 137.0 (C11); 146.8 (C13); 160.9 (C9); 165.6 (C14); 170.2 (C17) ppm; IR ν = 2951, 2862, 2360, 1723, 1139 cm^−1^; HRMS (ESI^+^) calcd *m/z* for C_36_H_52_N_4_O_8_ [M + H]^+^: 669.3858, found: 669.3853.

#### 3.2.15. Synthesis of 6,6′-[{2,2-dimethylcyclobutane-1,3-diylbis[(carboxymethyl) azane methylene diyl]}- bis(methylene)] dipicolinic acid (**25**)

A mixture containing compound **24** (200 mg, 0.30 mmol) and LiOH (45 mg, 1.19 mmoL) in 1:1 THF/H_2_O (5 mL) was stirred at room temperature for 4 h. Solvent was removed, the residue was poured into 4 M HCl in dioxane (8 mL) and the mixture was stirred for 20 h. Solvent was evaporated under vacuo, water (2 mL) was added and the resultant mixture was lyophilized to afford quantitatively tetraacid **25** (170 mg) as a brown solid. ^1^H NMR (250 MHz, D_2_O) δ = 0.78 (s, 3H, H5/6), 0.97 (s, 3H, H5/6), 1.78 (m, 1H, H4), 2.10 (m, 1H, H1/3/4), 2.30 (m, 2H, H1/3/4), 3.32 (m, 4H, H7), 3.98 (s, 4H, H8/16), 4.57 (s, 4H, H8/16), 7.62 (d, *J* = 7.6 Hz, 2H, H10), 8.00 (t, *J* = 7.7 Hz, 2H, H11), 8.09 (d, *J* = 7.7 Hz, 2H, H12) ppm; ^13^C NMR (62.5 MHz, D_2_O) δ = 14.8 (C5,6); 27.4 (C4); 29.5 (C5,6); 36.7 (C1/3); 41.0 (C2); 54.1 (C7); 56.2 (C16); 27.8 (C8); 125.4 and 127.8 (C10/12); 139.8 (C11); 146.2 (C13); 149.1 (C9); 166.8 and 167.7 (C14/17) ppm; IR ν = 3363, 2360, 1730, 1630, 1400 cm^−1^; HRMS (ESI^+^) calcd m/z for C_26_H_32_N_4_O_8_ [M + H]^+^: 529.2293, found: 529.2283.

## 4. Conclusions

In this work, we showed the suitability of several cyclobutane scaffolds for the preparation of different families of products with potential usefulness in the fields of surfactants, gelators and metal cation ligands. Enantiomerically pure *cis*-1,2-disubstitued cyclobutane derivatives have been efficient and selectively obtained from a common chiral half-ester. From this precursor, synthetic routes to amphiphiles as possible surfactants have been achieved through a *cis*-β-CBAA as a convenient intermediate. In addition, compounds with appropriate structures to behave as gelators have been synthesized from an orthogonally protected *trans*-β-CBAA. Otherwise, symmetric *cis*-1,3-cyclobutane scaffolds have been prepared from verbenone through norpinic acid that provided a *cis*-1,5-diamine, which is a key intermediate in the synthesis of different poly(amino acids). These compounds are valuable candidates to be investigated as polydentate ligands for lanthanide complexes. The properties of these families of compounds as well as their likely applications are the subject of active investigation in our laboratory.

## Supplementary Materials

Supplementary materials can be found at https://www.mdpi.com/1422-0067/20/18/4333/s1.

## Abbreviations

ATR	Attenuated Total Reflection
CBAA	Cyclobutane Amino Acid
DDS	Drug Delivery Systems
DIPEA	Diisopropylethylamine
DMAP	Dimethylaminopyridine
DMF	Dimethylformamide
DNA	Deoxyribonucleic Acid
ESI	Electrospray Ionization
HRMS	High-Resolution Mass Spectrometry
IR	Infrared
LMWG	Low Molecular-Weight Gelator
MS	Mass Spectrometry
NMR	Nuclear Magnetic Resonance
NPY	Neuropeptide Y
PyBOP	(Benzotriazol-1-yloxy)tripyrrolidinophosphonium hexafluorophosphate
QTOF	Quadrupole Time-of-Flight
TEA	Triethylamine
TFA	Trifluoroacetic Acid
THF	Tetrahydrofuran
TLC	Thin layer chromatography
