# Supplementary material for: Cyclobutane-Containing Scaffolds as Useful Intermediates in the Stereoselective Synthesis of Suitable Candidates for Biomedical Purposes: Surfactants, Gelators and Metal Cation Ligands
Source: Int J Mol Sci. 2019 Sep 4;20(18):4333. doi: 10.3390/ijms20184333 (PMC6770955; doi:10.3390/ijms20184333)

SUPPLEMENTARY MATERIALS

Cyclobutane-containing Scaffolds as Useful Intermediates in the Stereoselective Synthesis of Suitable Candidates for Biomedical Purposes: Surfactants, Gelators and Metal Cation Ligands

Ona Illa,* Albert Serra, Agustí Ardiaca, Xavier Herrero, Guillem Closa, and Rosa M. Ortuño*

^{1}H and ^{13}C NMR spectra of compounds **2, 3, 5, 6, 8, 9, 11-14, 16, 17, 20, 21, 24** and **25**

^{1}H-NMR (250 MHz, $CDCl_3$) of compound **2**

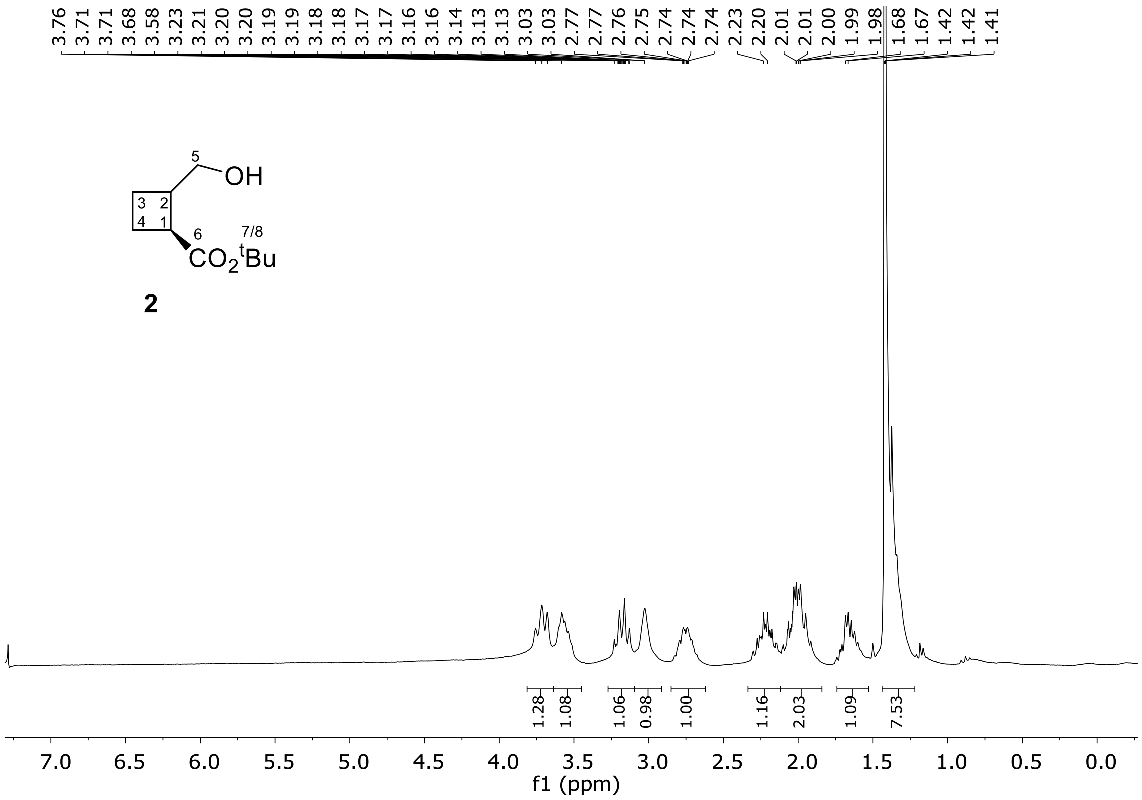

^{13}C-NMR (62.5 MHz, $CDCl_3$) of compound **2**

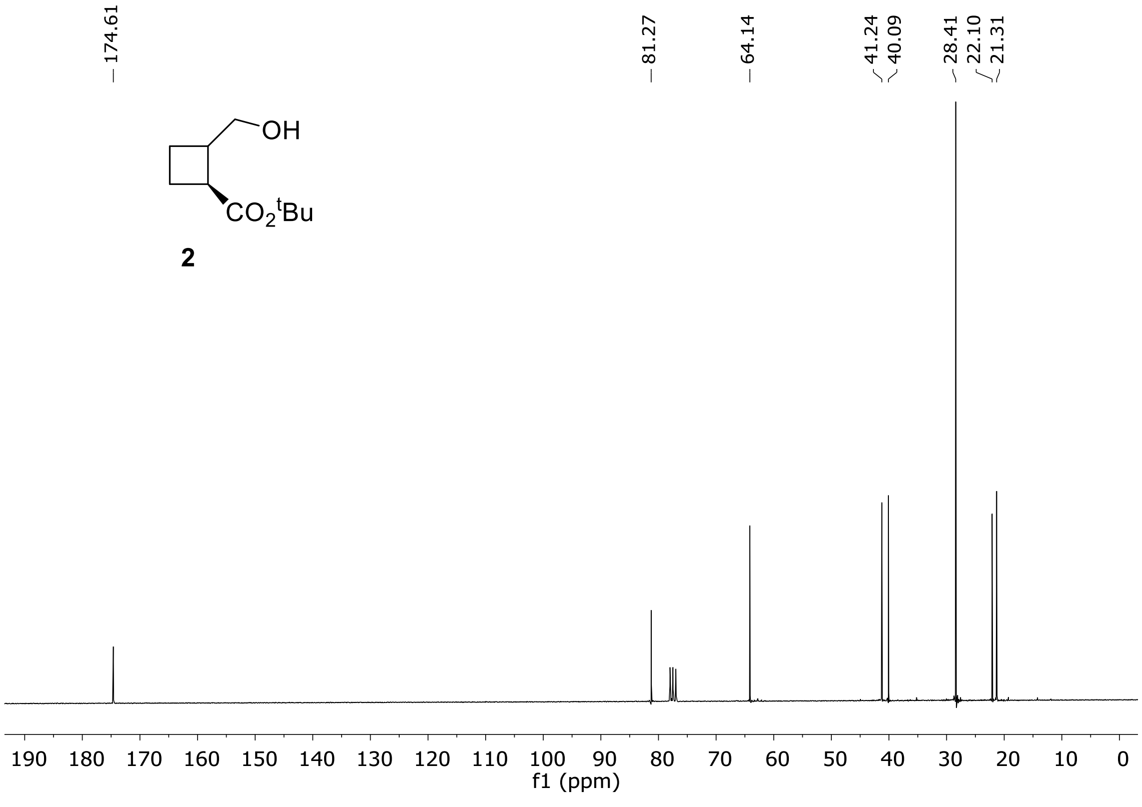

^{1}H-NMR (360 MHz, $CDCl_3$) of compound **3**

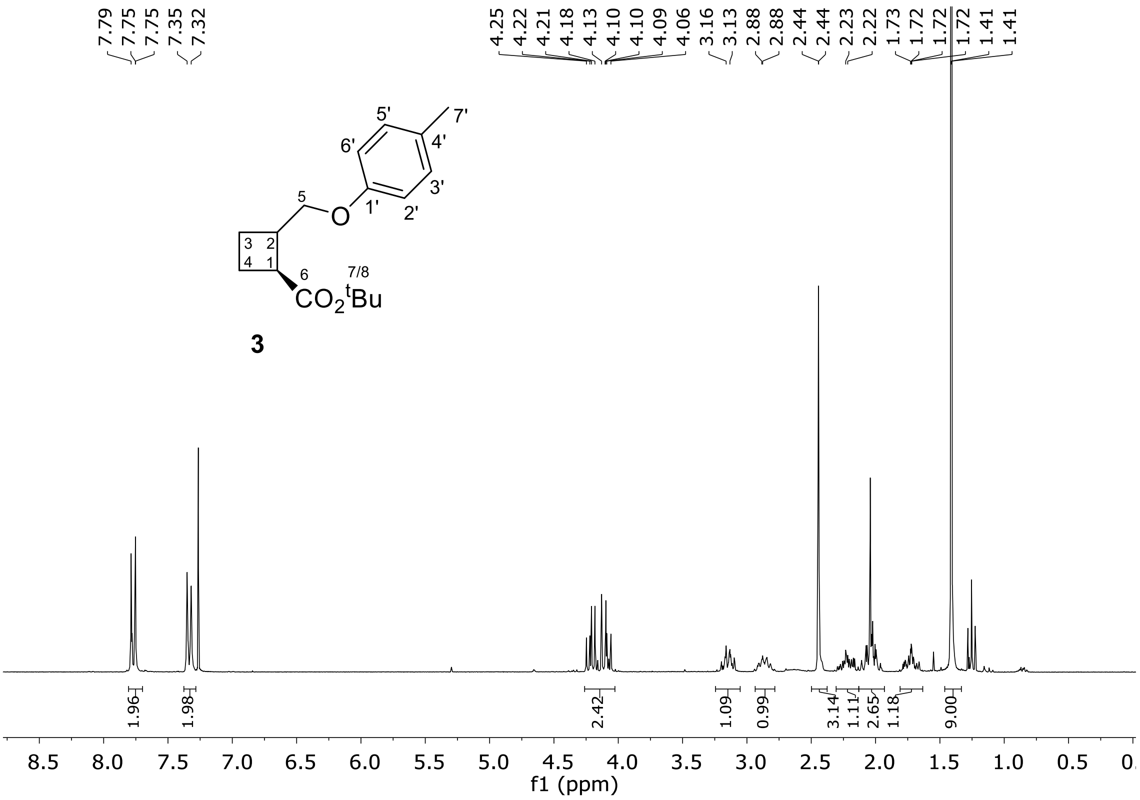

^{13}C-NMR (90 MHz, $CDCl_3$) of compound **3**

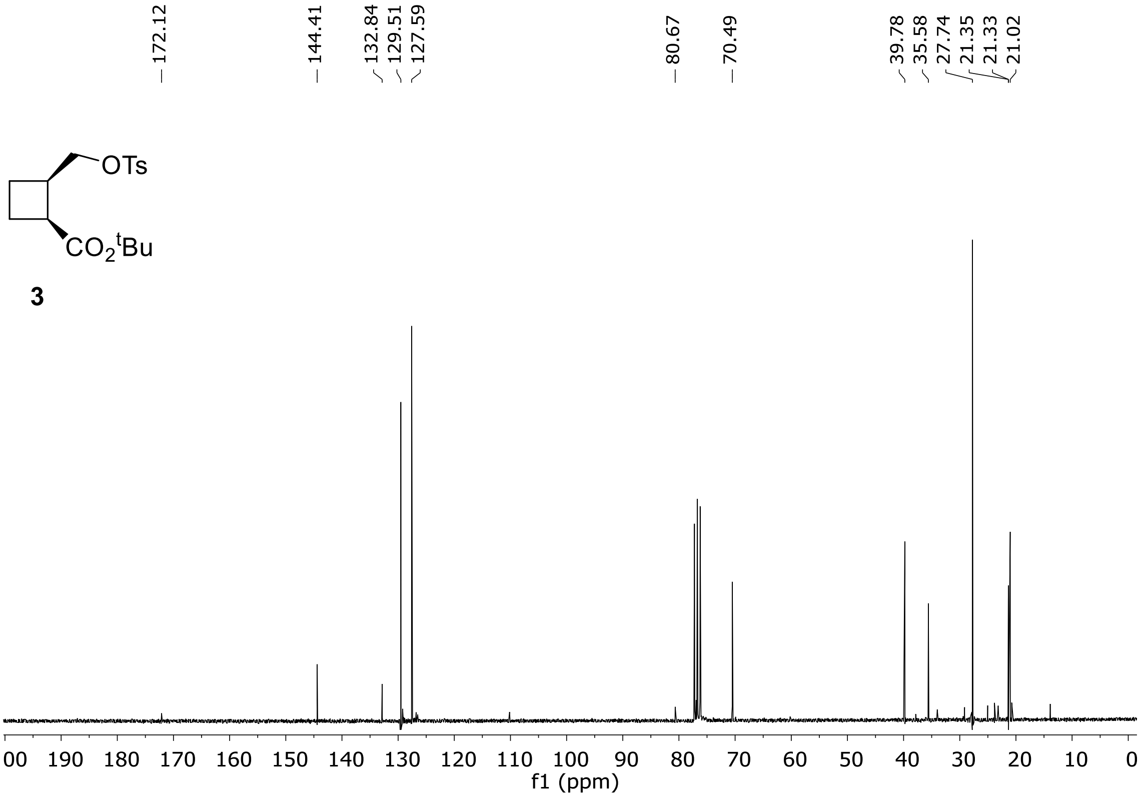

^{1}H-NMR (250 MHz, $CDCl_3$) of compound **5**

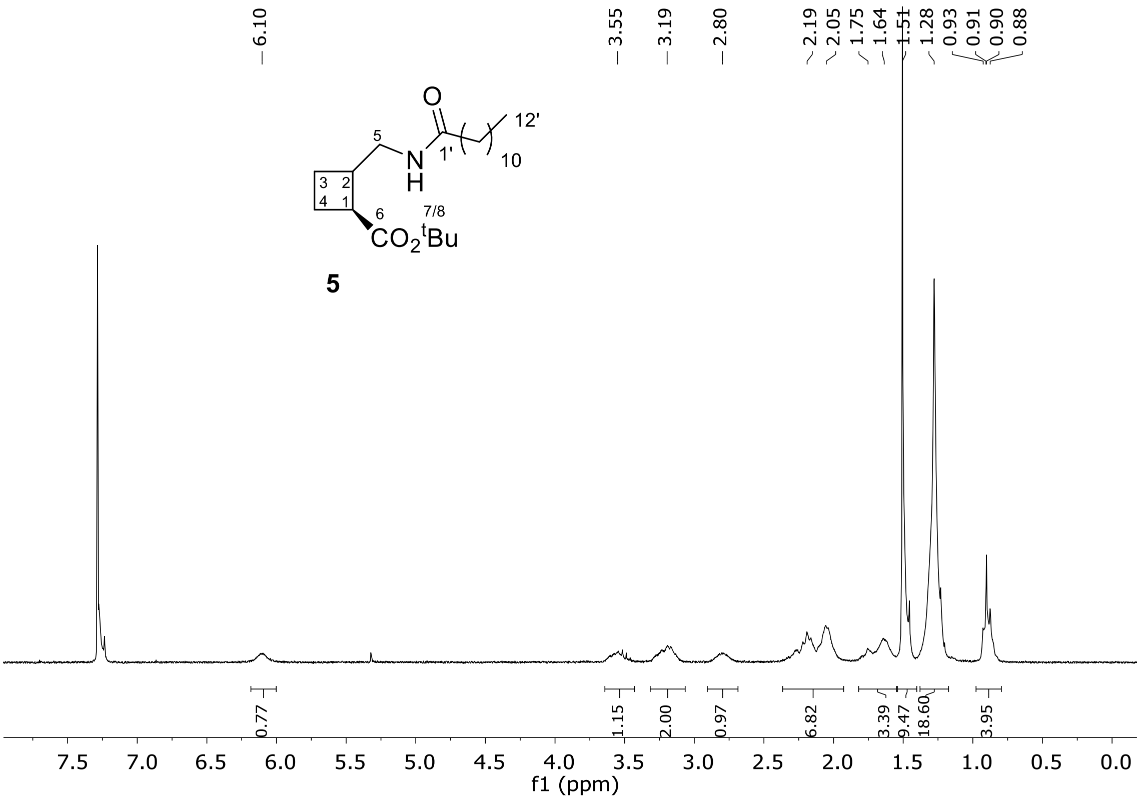

^{13}C-NMR (62.5 MHz, $CDCl_3$) of compound **5**

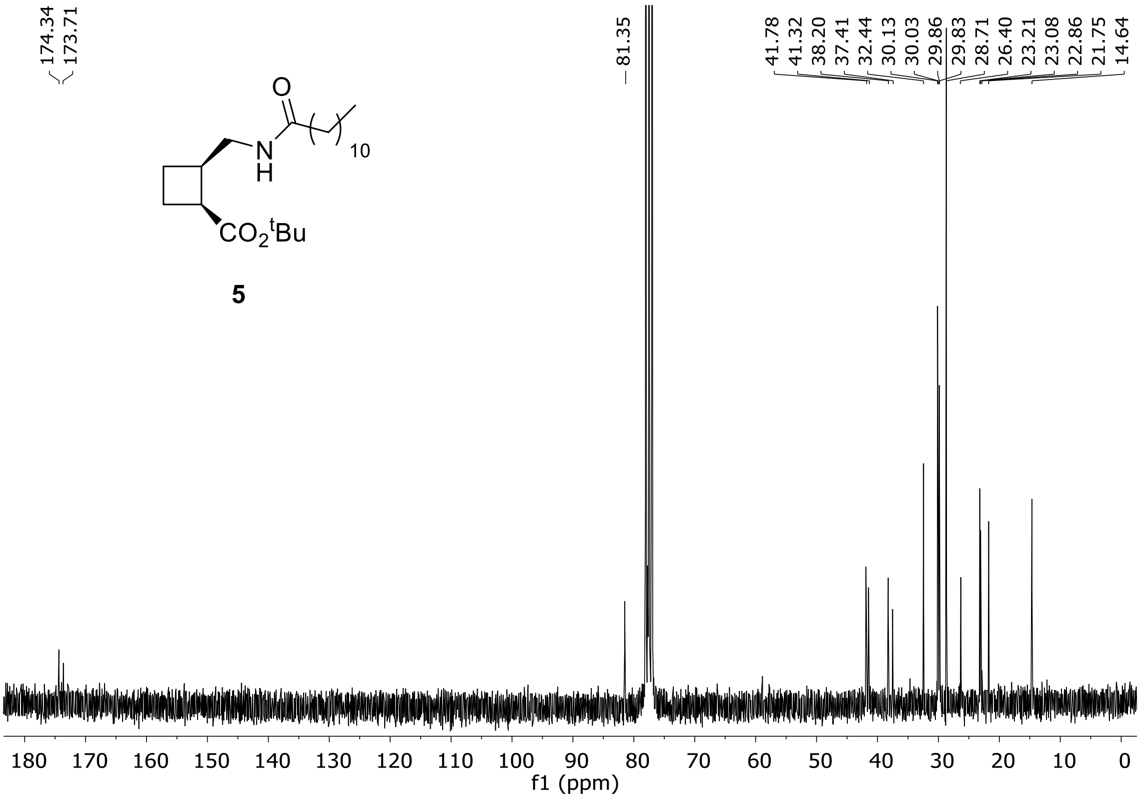

^{1}H-NMR (250 MHz, $CDCl_3$) of compound **6**

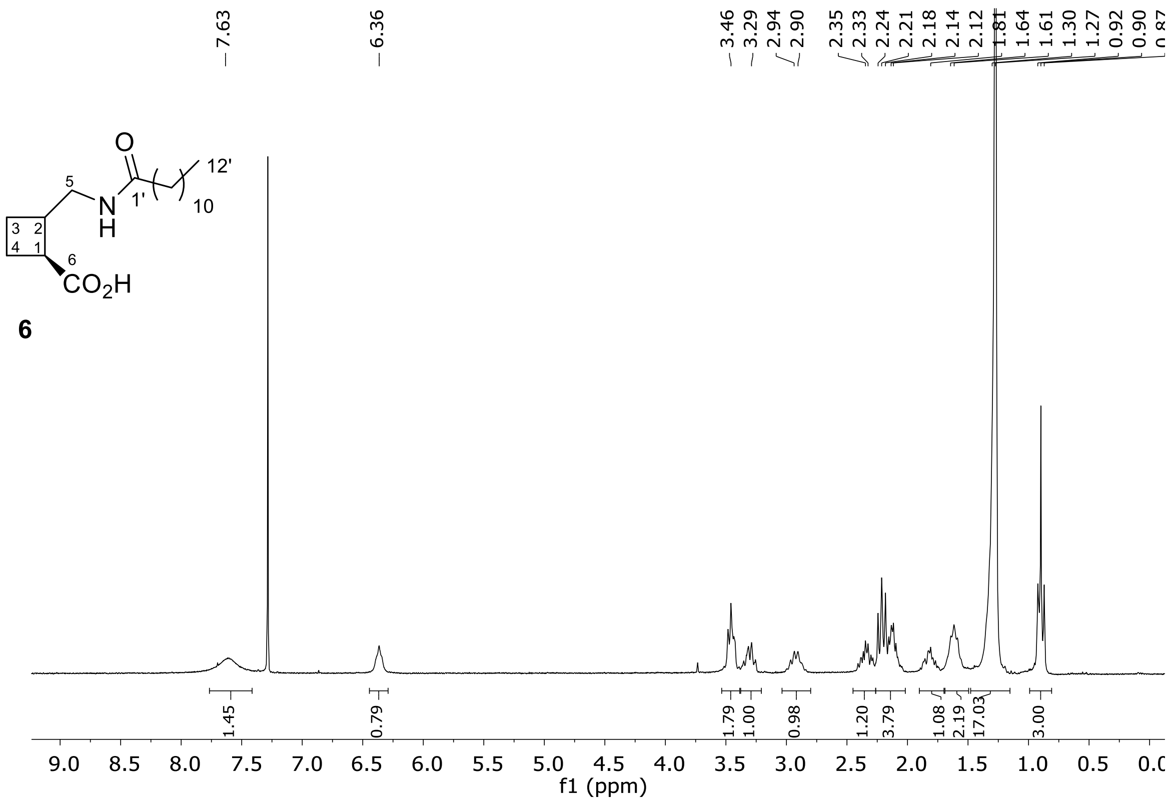

^{13}C-NMR (62.5 MHz, $CDCl_3$) of compound **6**

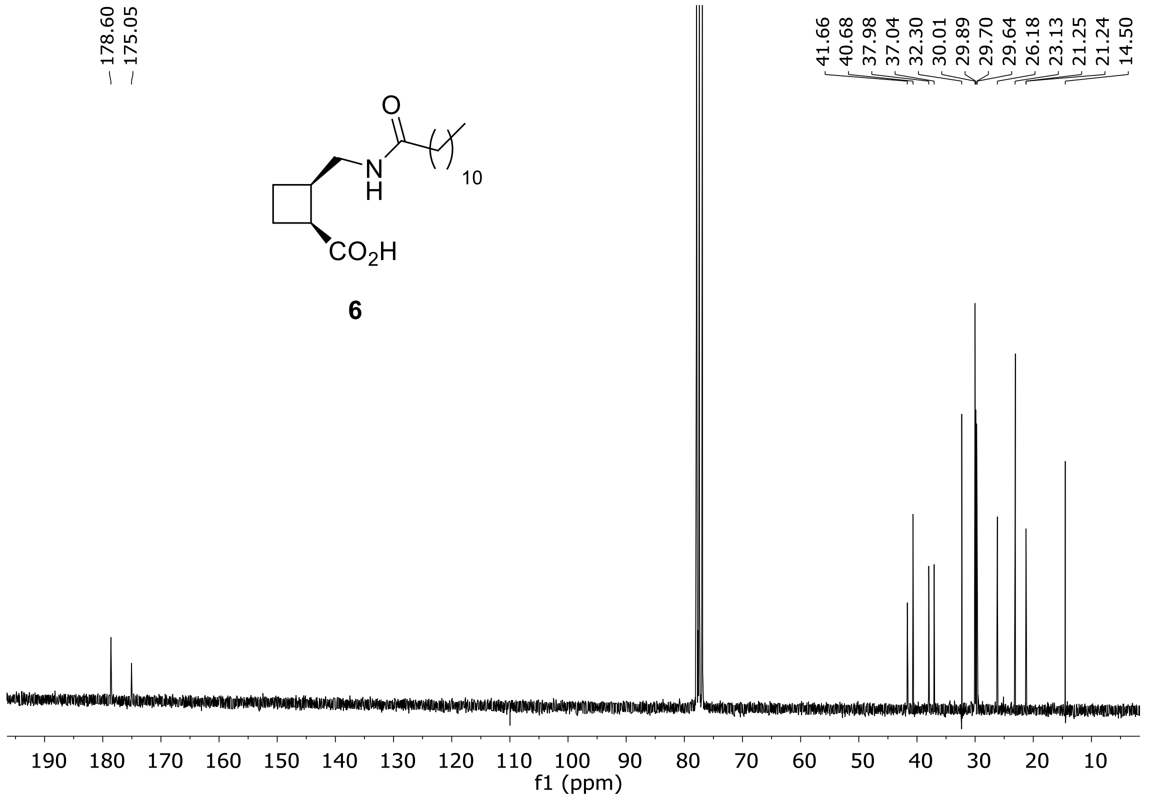

^{1}H-NMR (250 MHz, $CDCl_3$) of compound **8**

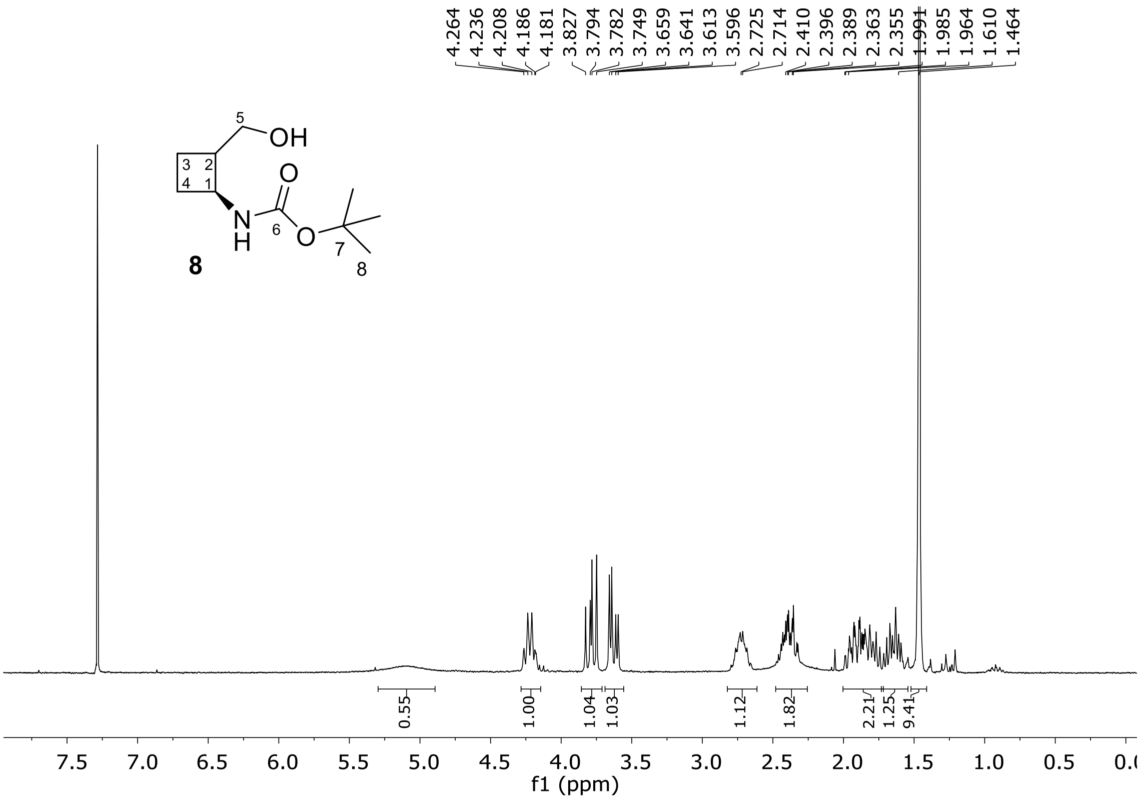

^{13}C-NMR (62.5 MHz, $CDCl_3$) of compound **8**

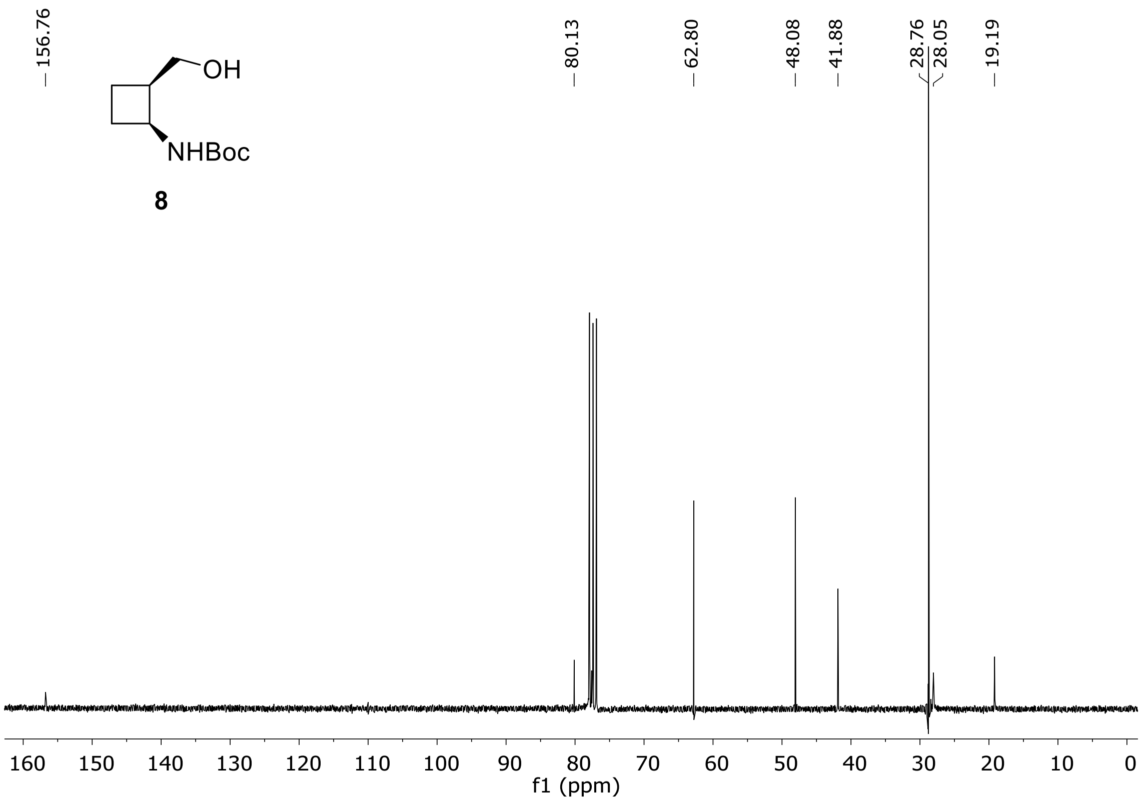

^{1}H-NMR (360 MHz, $CDCl_3$) of compound **9**

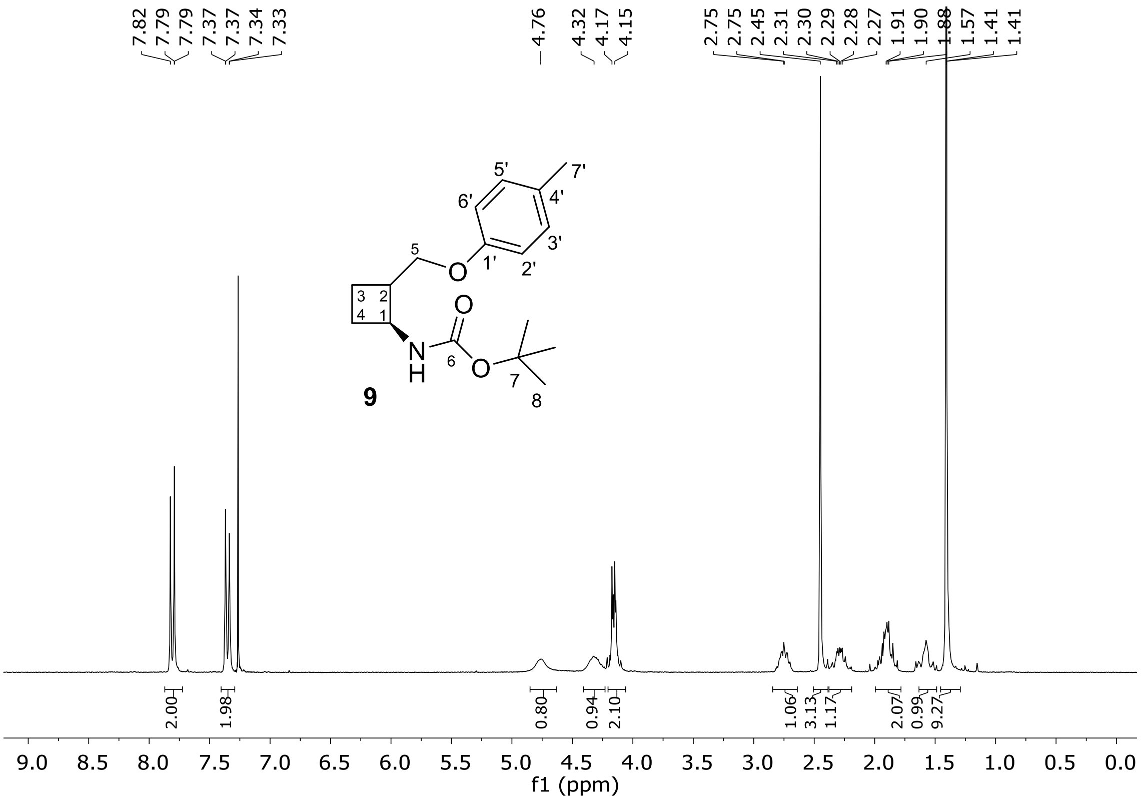

^{13}C-NMR (90 MHz, $CDCl_3$) of compound **9**

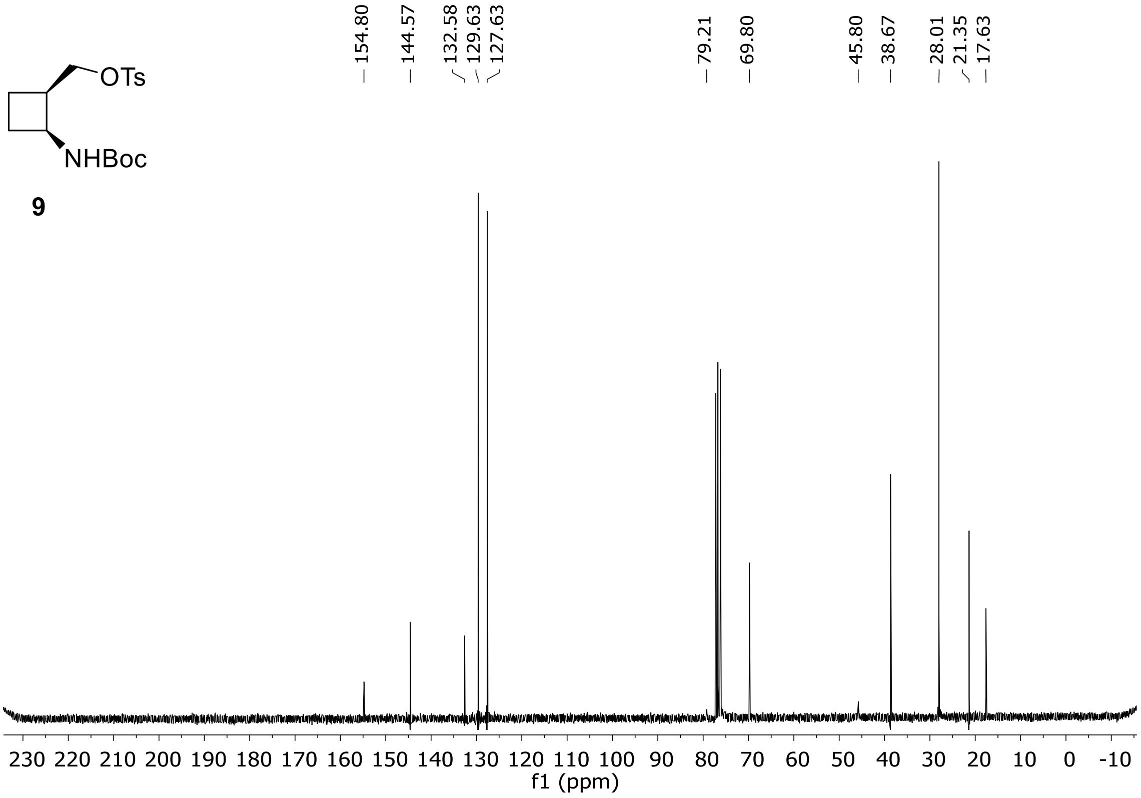

^{1}H-NMR (360 MHz, $CDCl_3$) of compound **11**

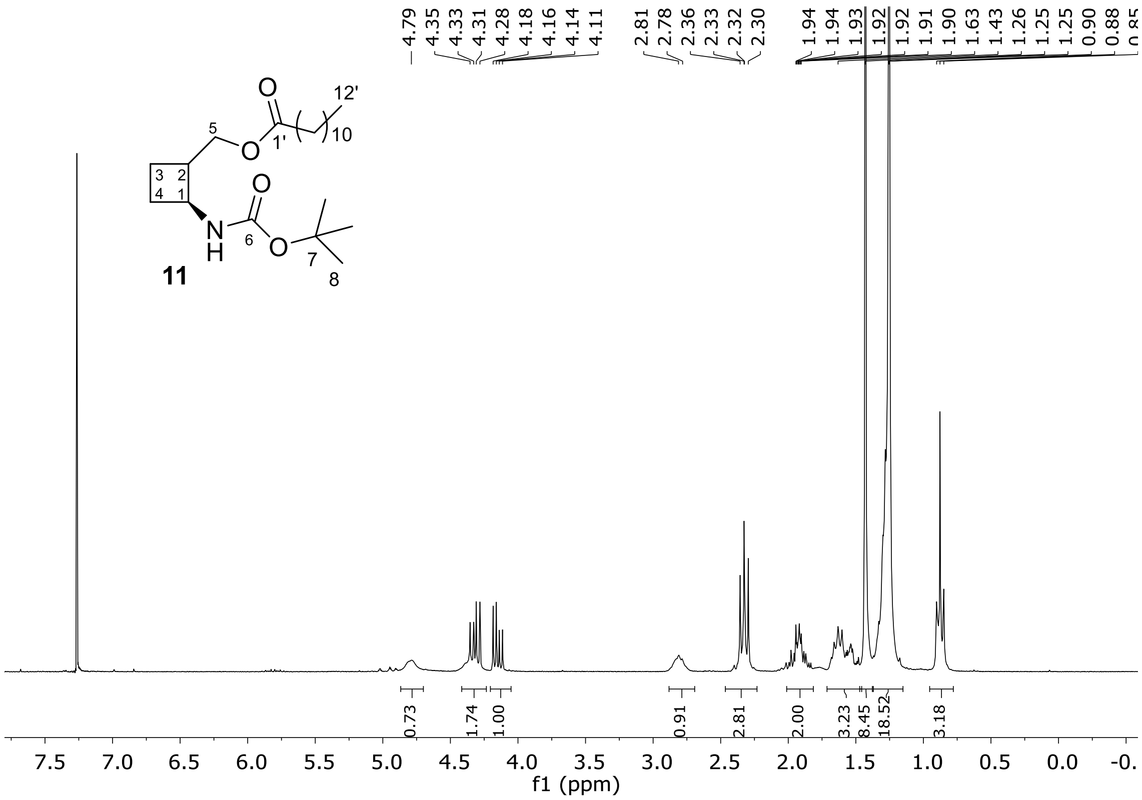

^{13}C-NMR (90 MHz, $CDCl_3$) of compound **11**

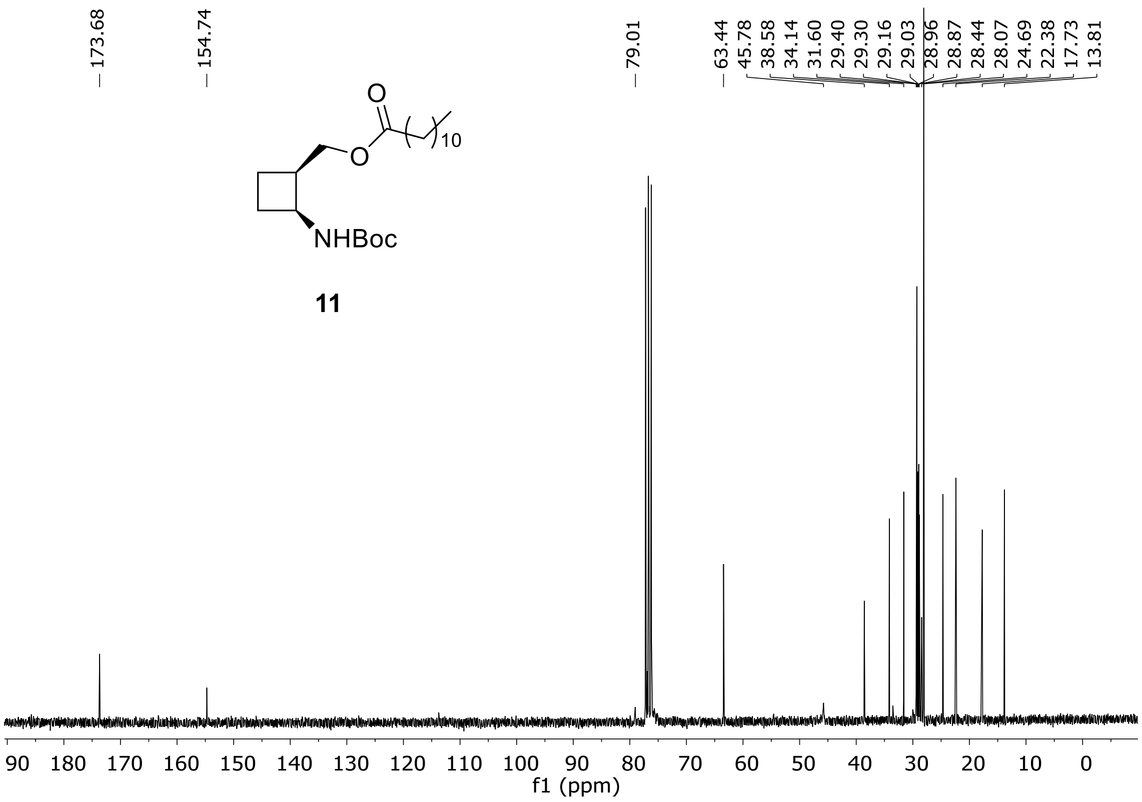

^{1}H-NMR (360 MHz, CD_3OD) of compound **12**

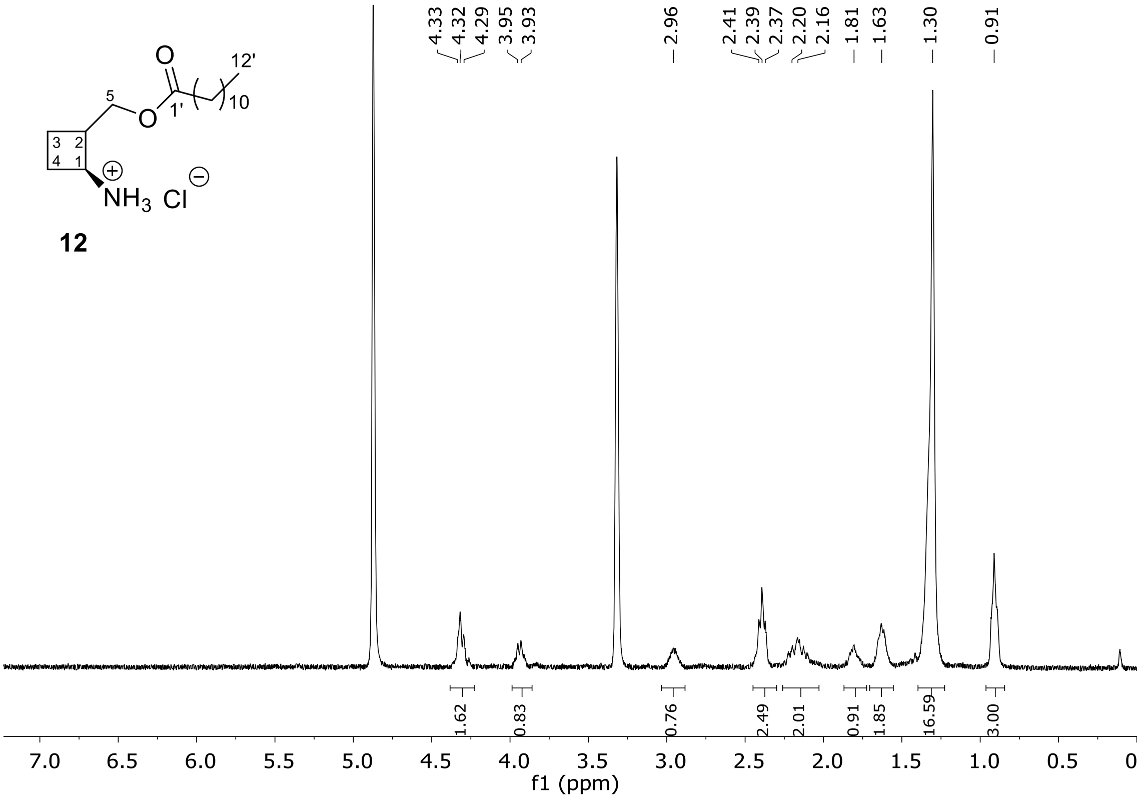

^{13}C-NMR (90 MHz, CD_3OD) of compound **12**

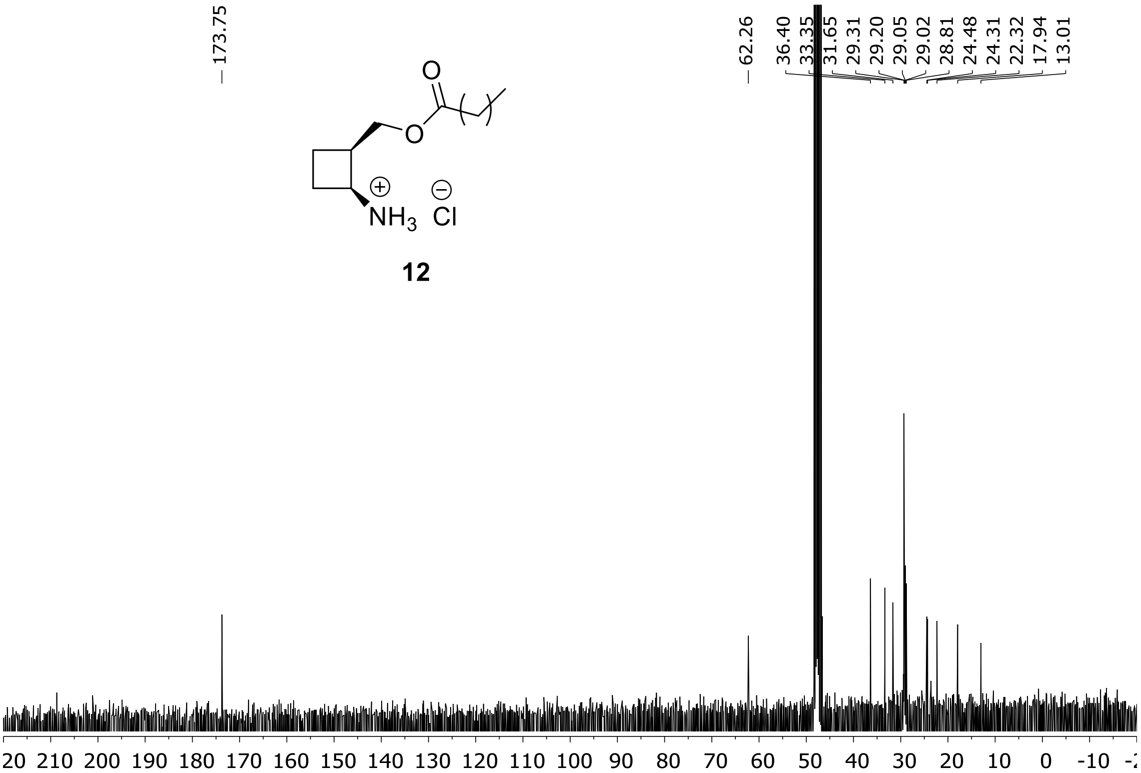

^{1}H-NMR (360 MHz, $CDCl_3$) of compound **13**

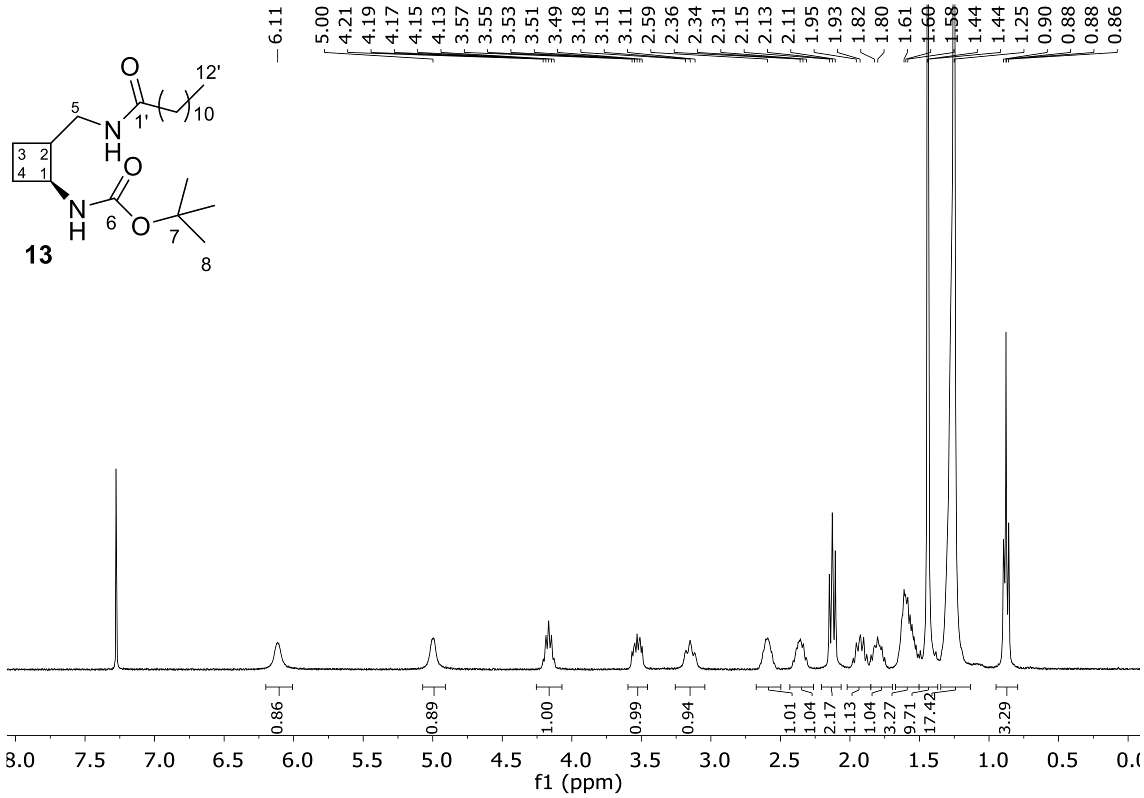

^{13}C-NMR (90 MHz, $CDCl_3$) of compound **13**

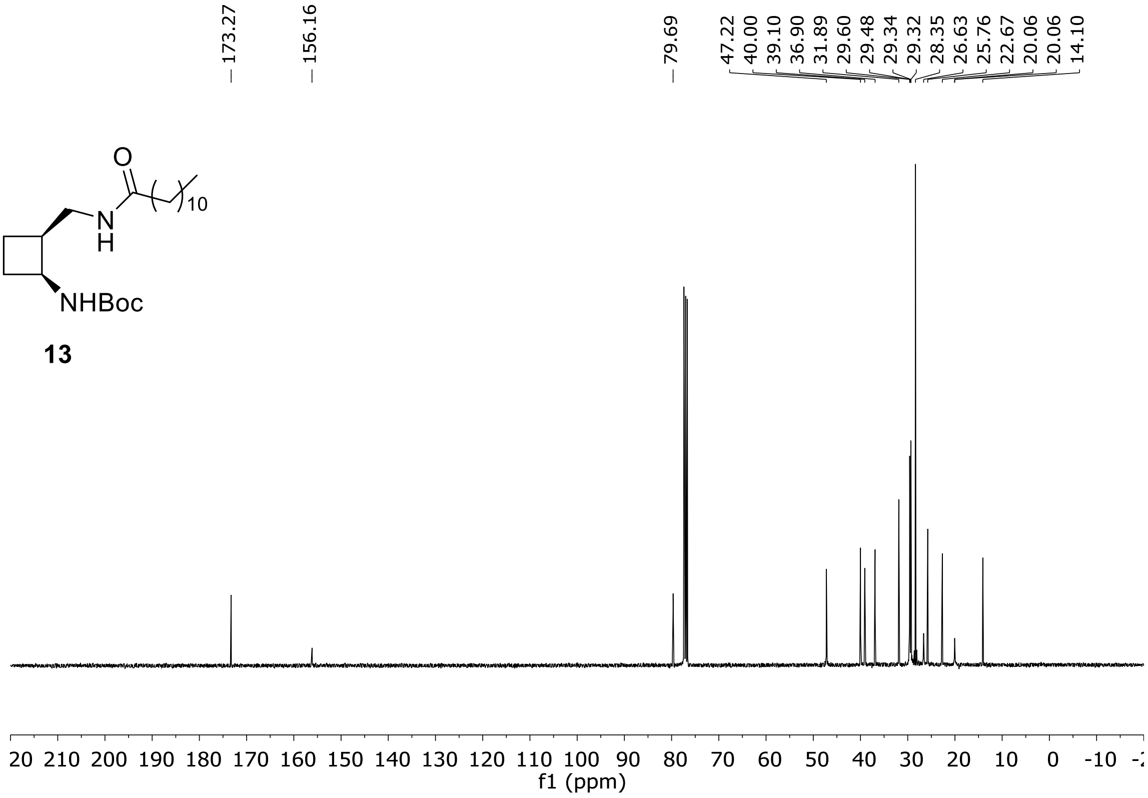

^{1}H-NMR (360 MHz, CD_3OD) of compound **14**

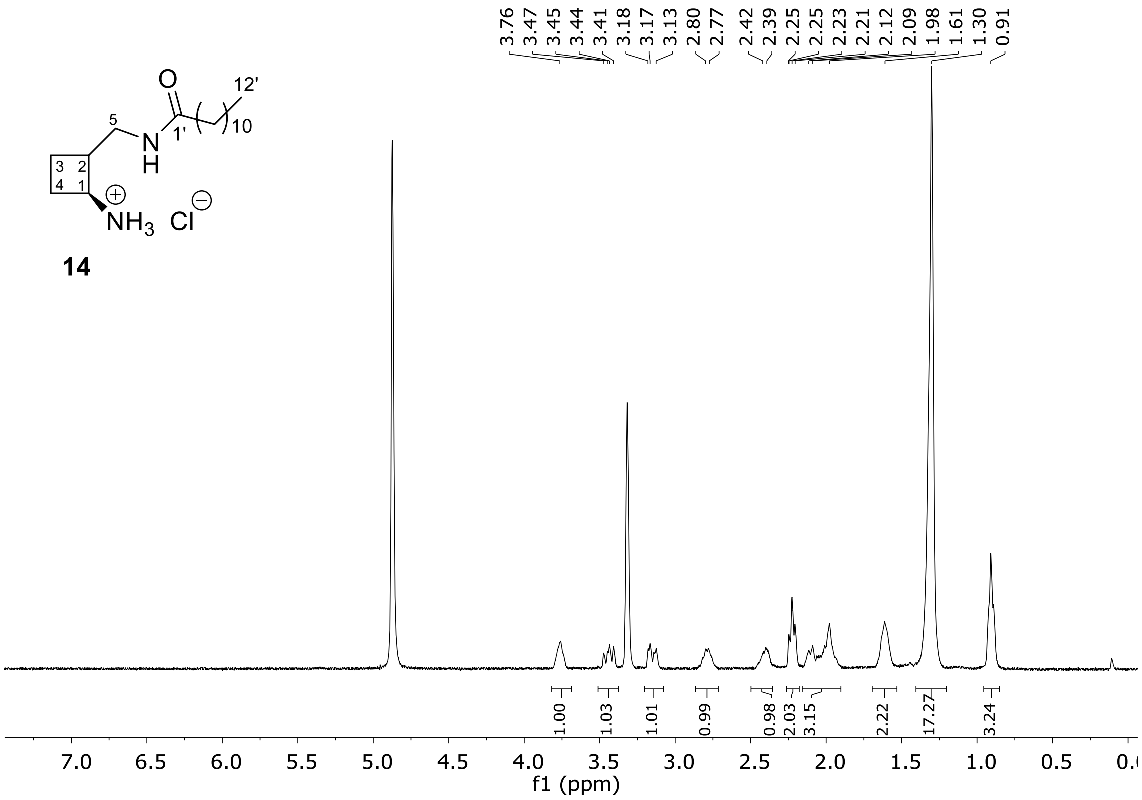

^{13}C-NMR (90 MHz, CD_3OD) of compound **14**

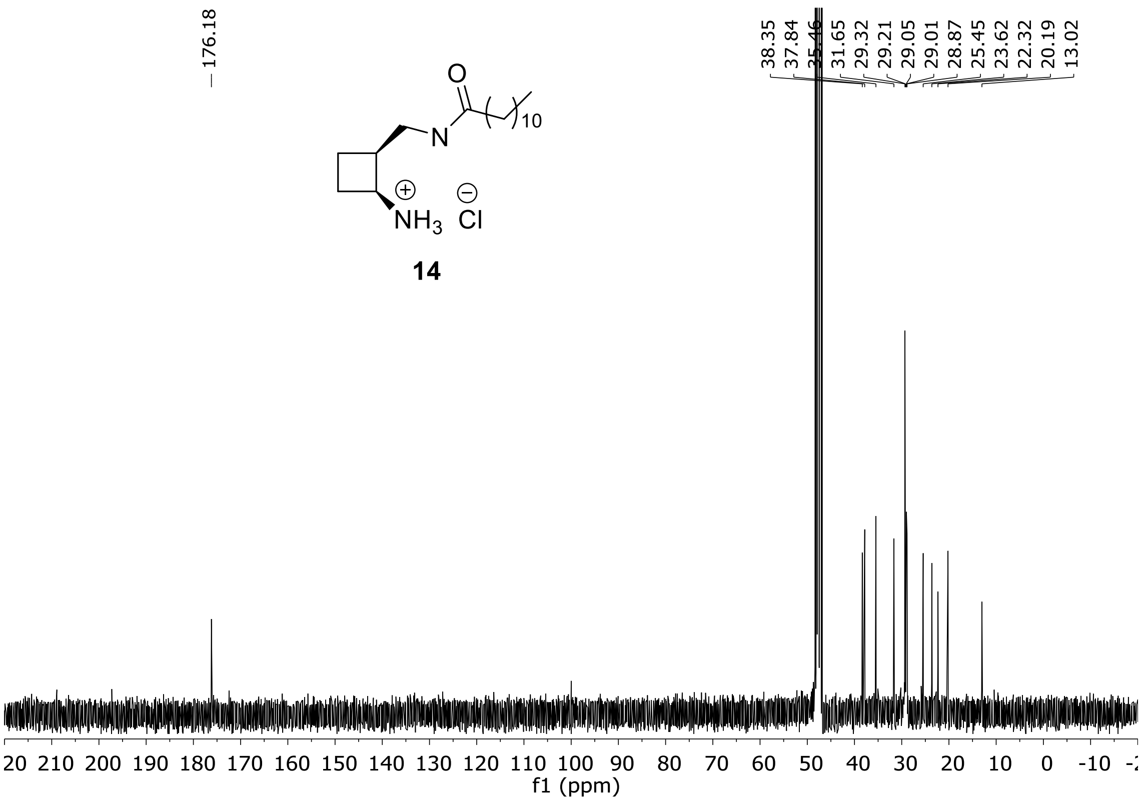

^{1}H-NMR (360 MHz, $CDCl_3$) of compound **16**

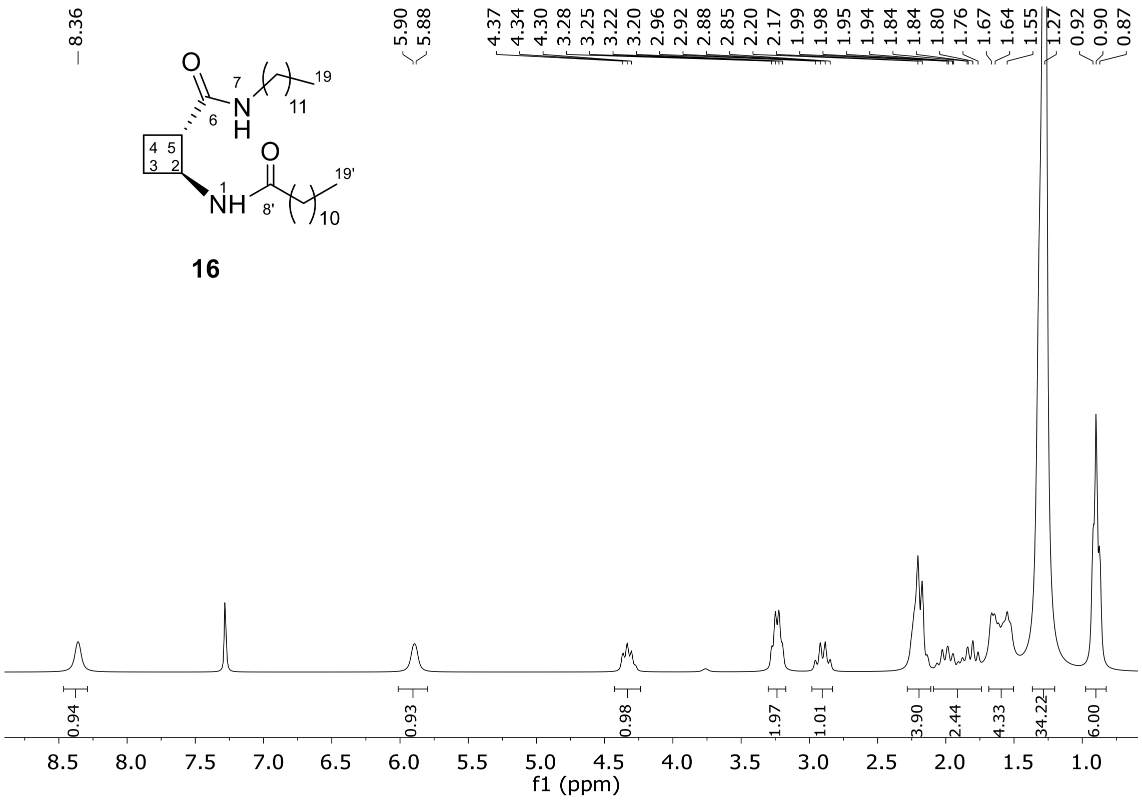

^{13}C-NMR (62.5 MHz, $CDCl_3$) of compound **16**

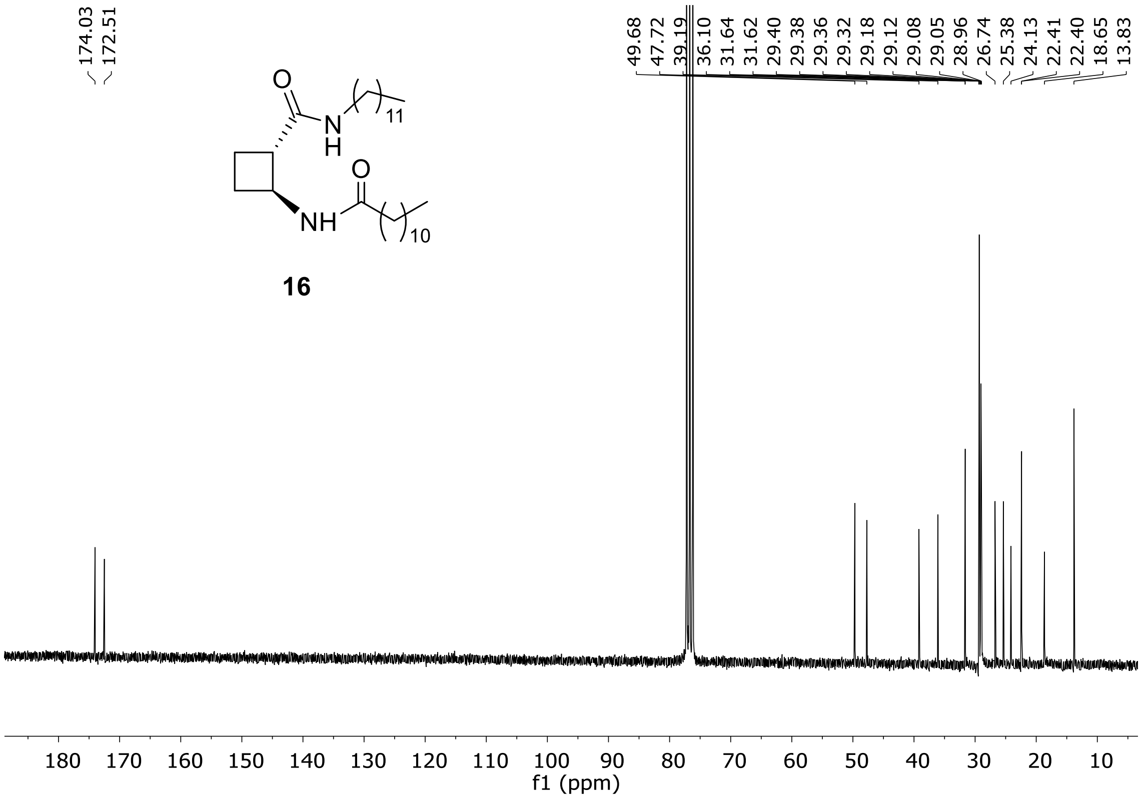

^{1}H-NMR (250MHz, $CDCl_3$) of compound **17**

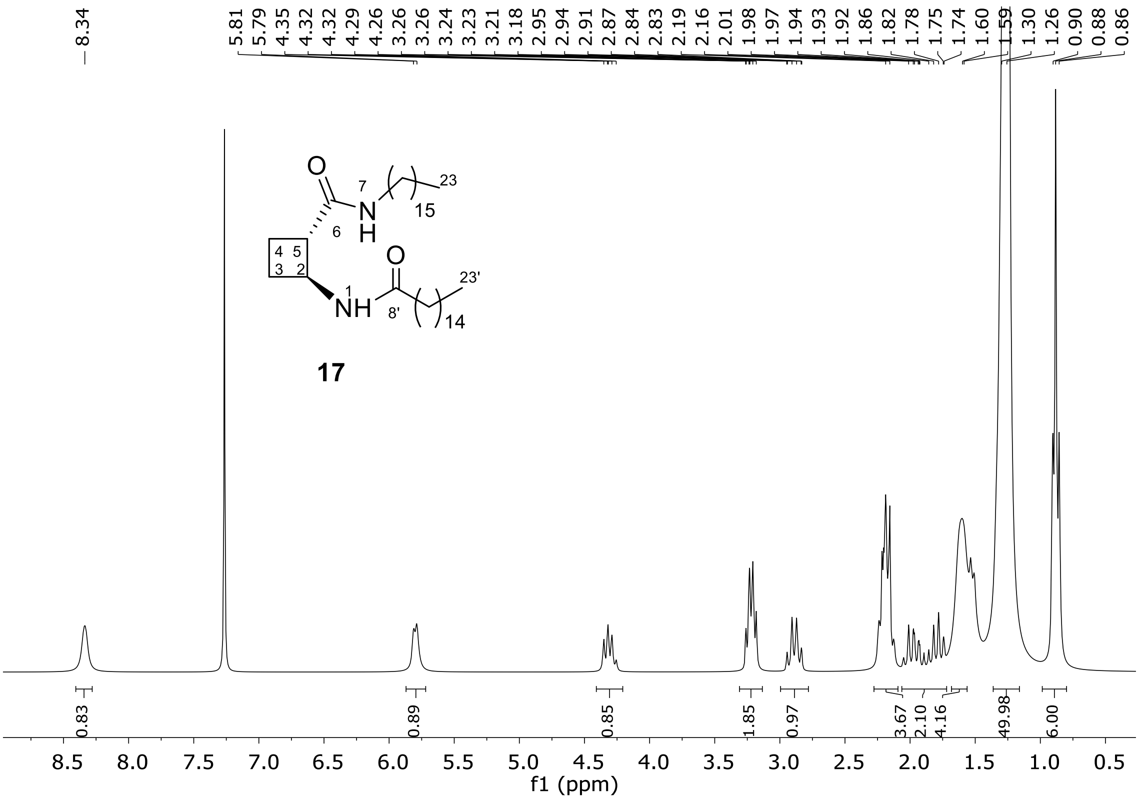

^{13}C-NMR (62.5 MHz, $CDCl_3$) of compound **17**

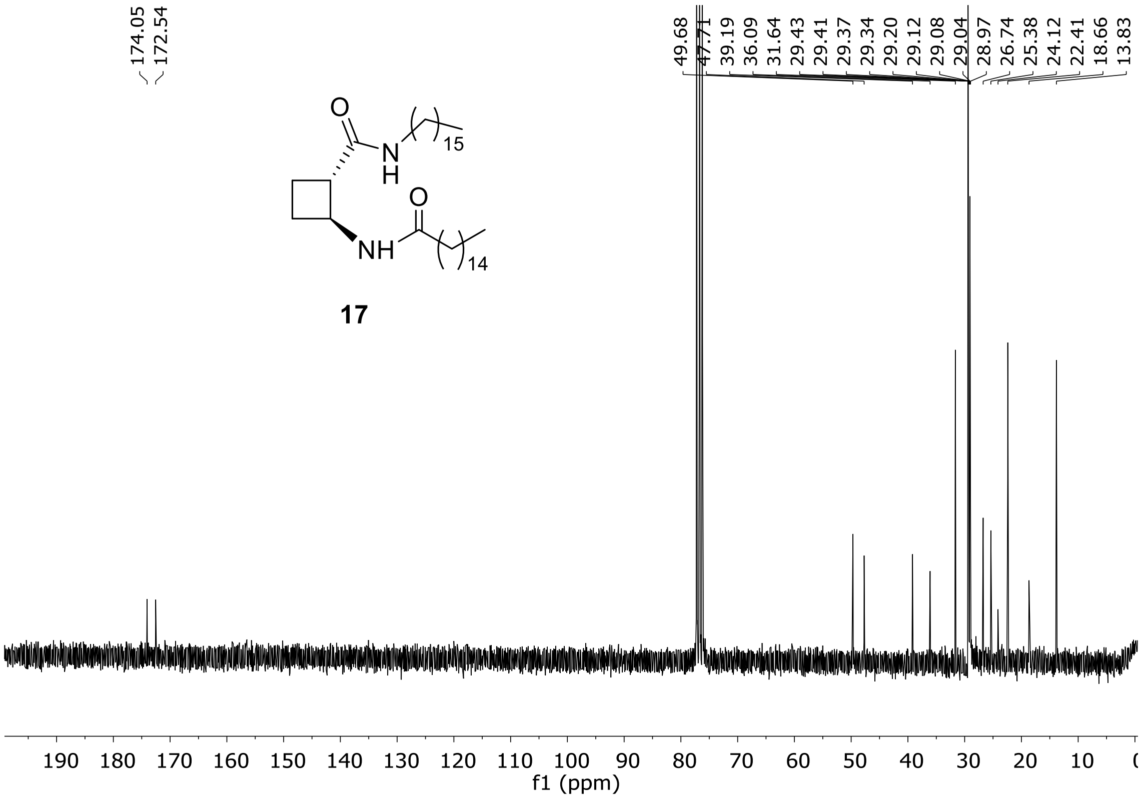

^{1}H-NMR (250 MHz, $CDCl_3$) of compound **20**

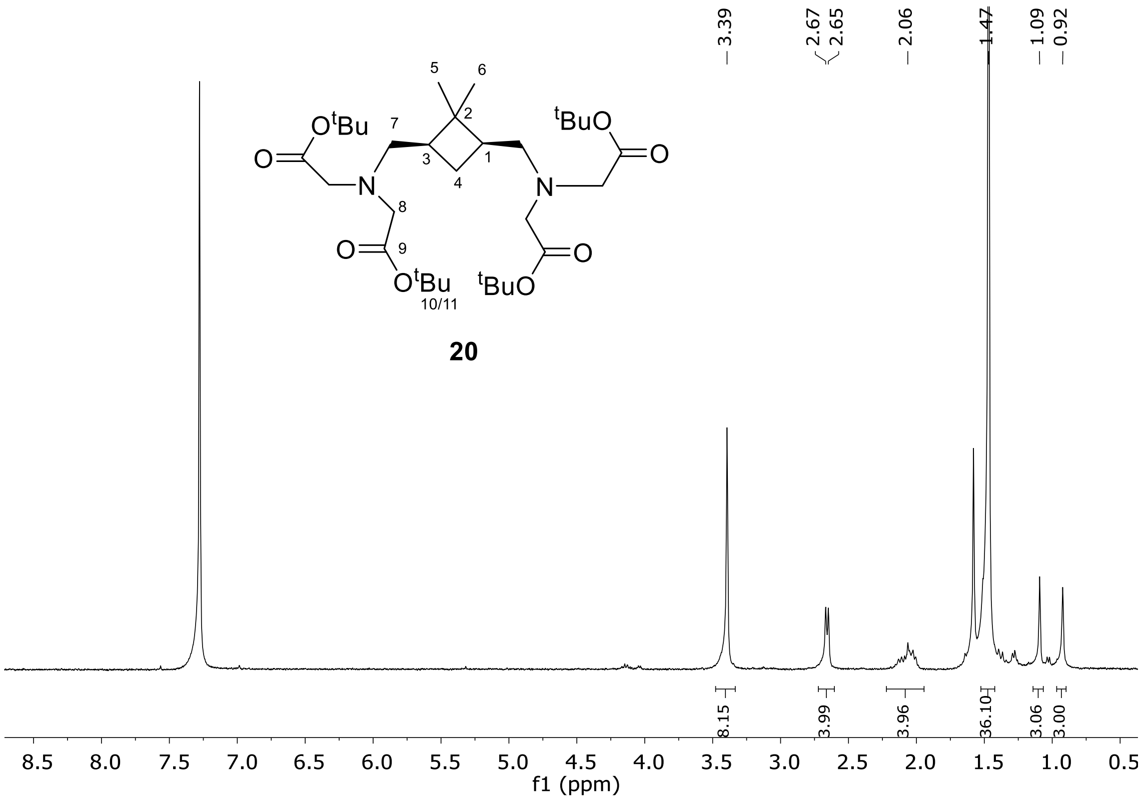

^{13}C-NMR (62.5 MHz, $CDCl_3$) of compound **20**

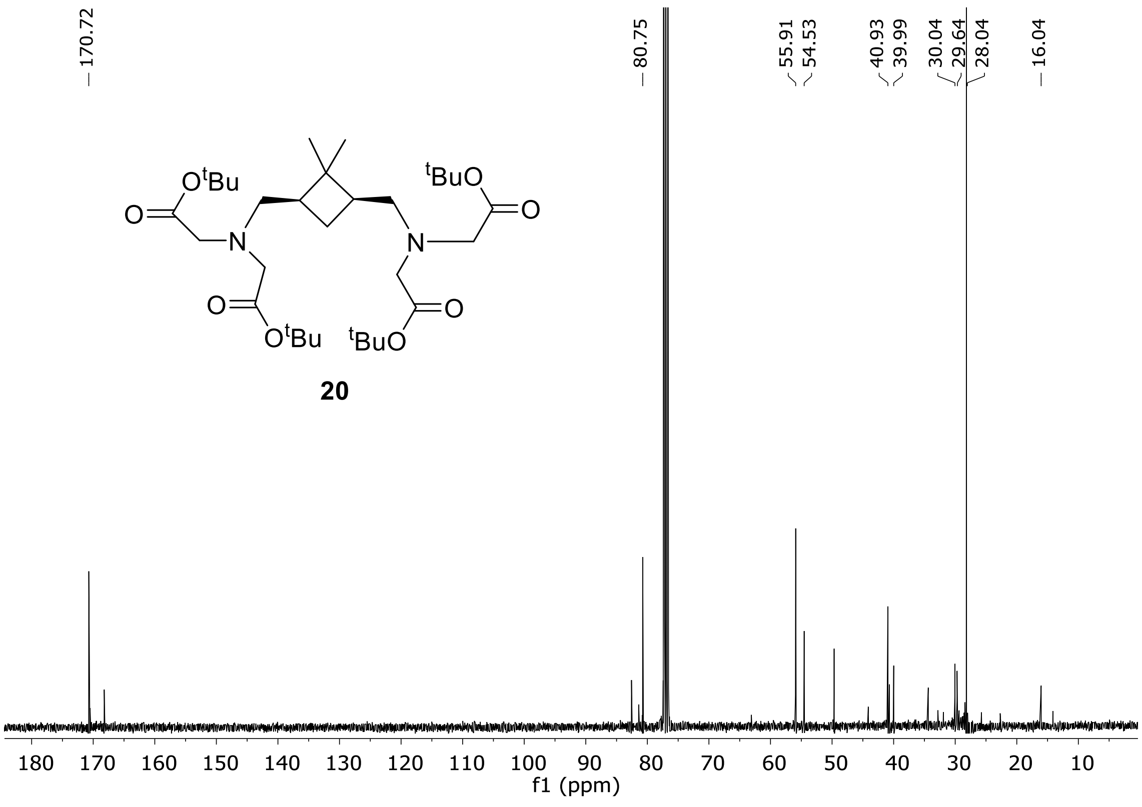

^{1}H-NMR (360 MHz, D_2O) of compound **21**

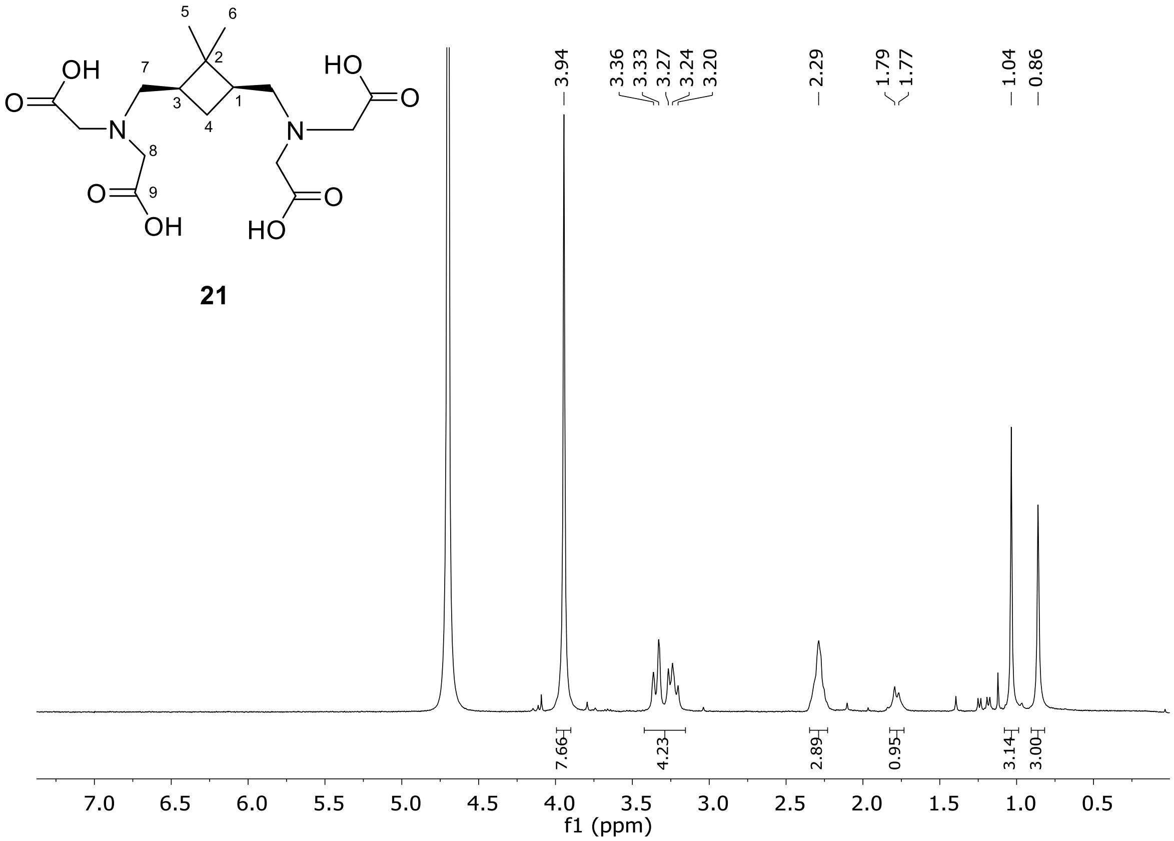

21

^{13}C-NMR (62.5 MHz, D_2O) of compound **21**

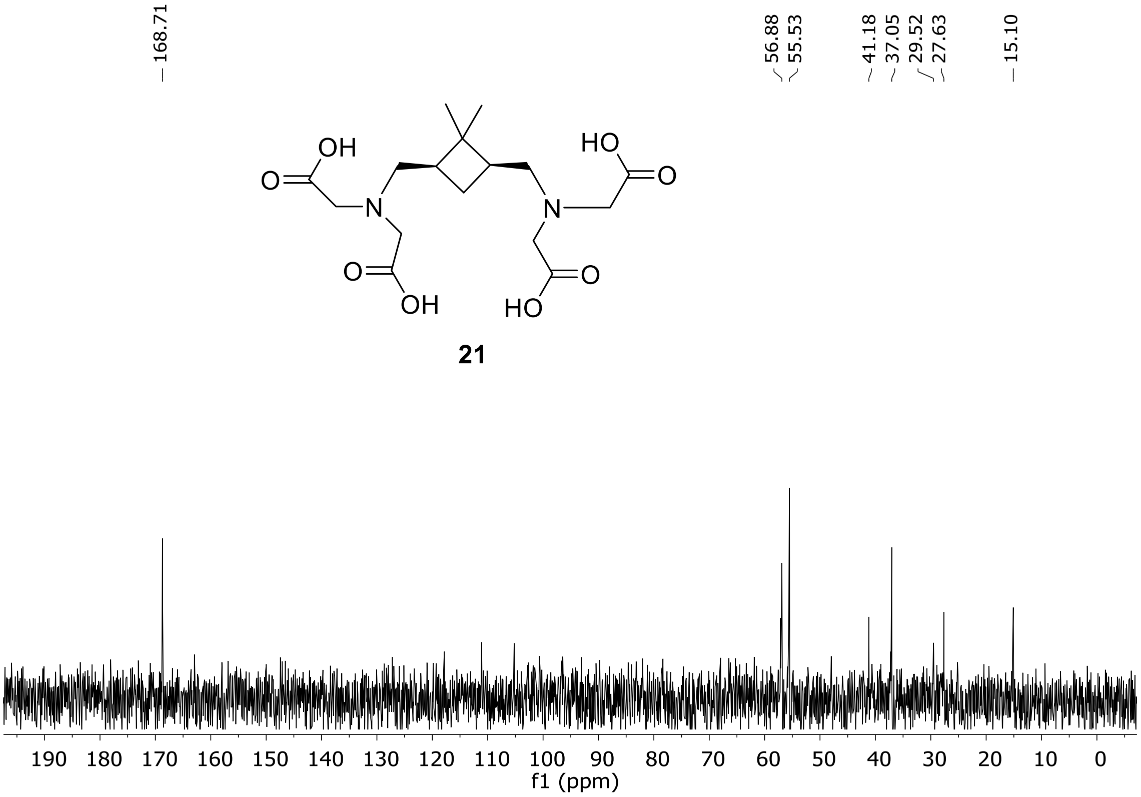

21

^{1}H-NMR (250 MHz, $CDCl_3$) of compound **23**

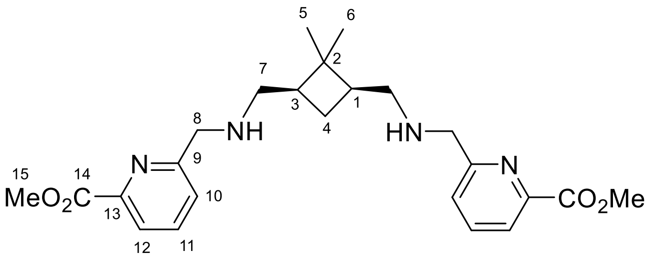

23

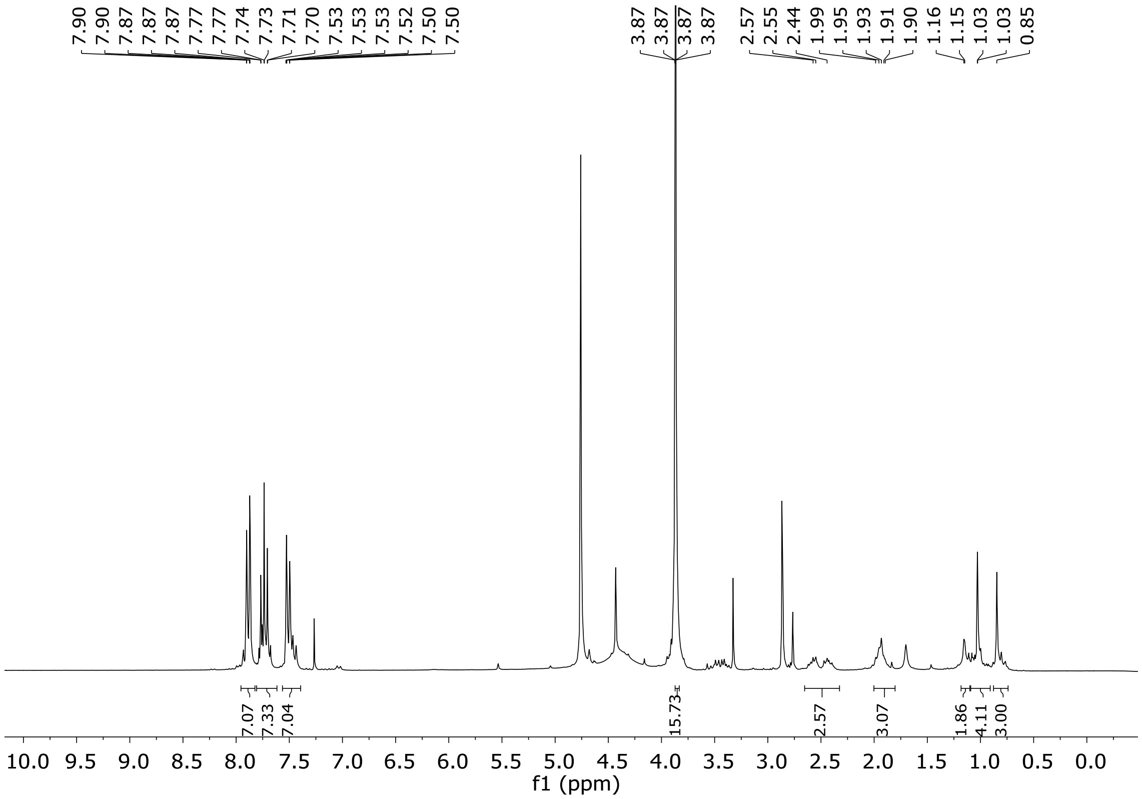

^{1}H-NMR (400 MHz, $CDCl_3$) of compound **24**

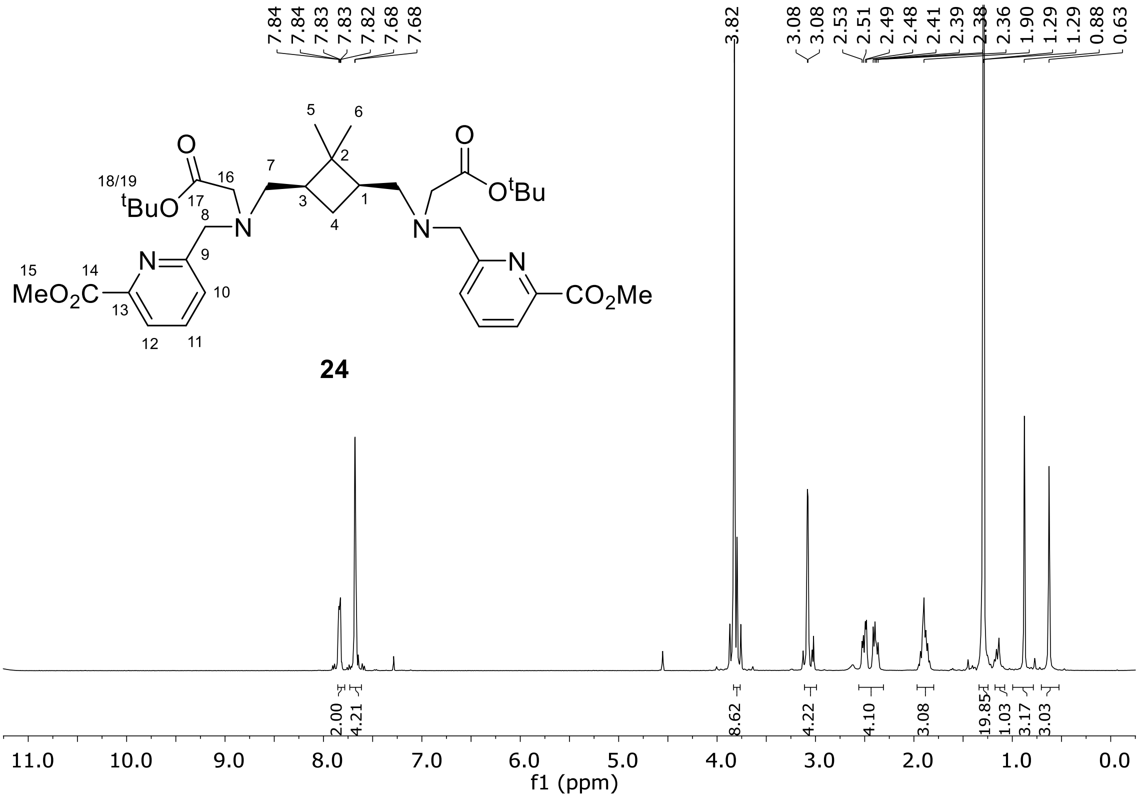

^{13}C-NMR (62.5 MHz, $CDCl_3$) of compound **24**

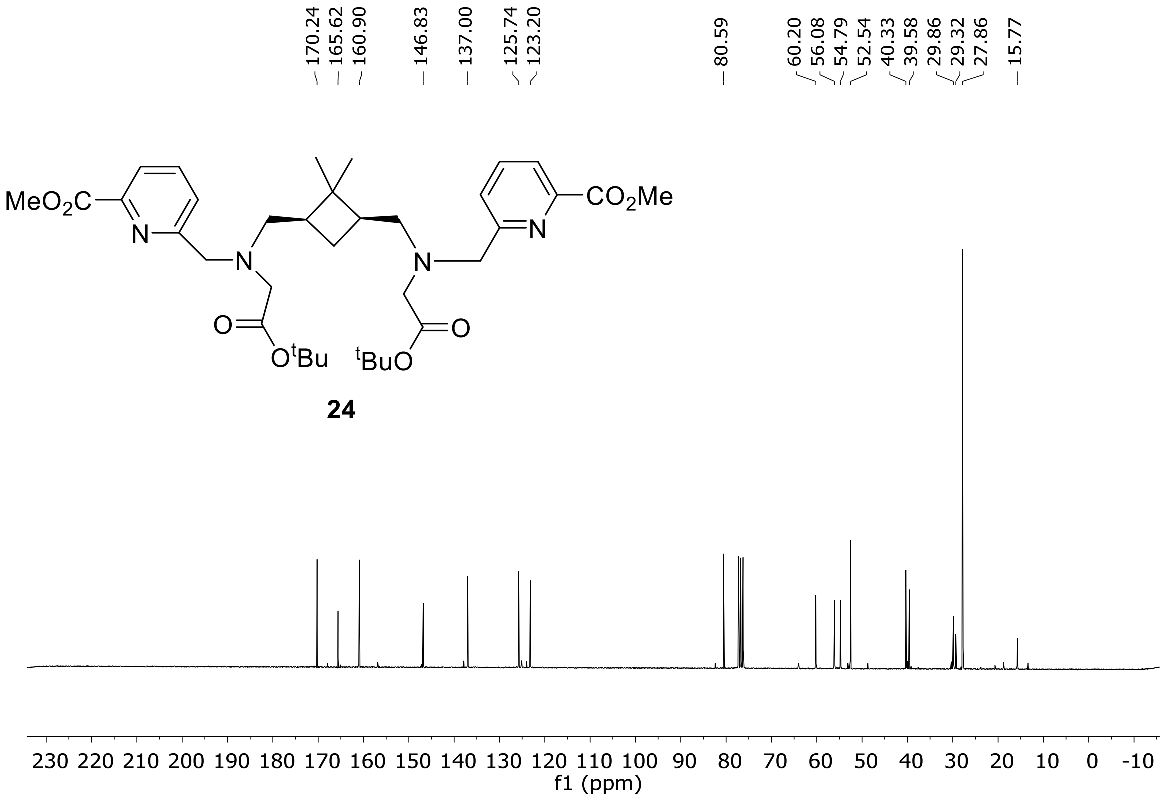

^{1}H-NMR (250 MHz, D_2O) of compound **25**

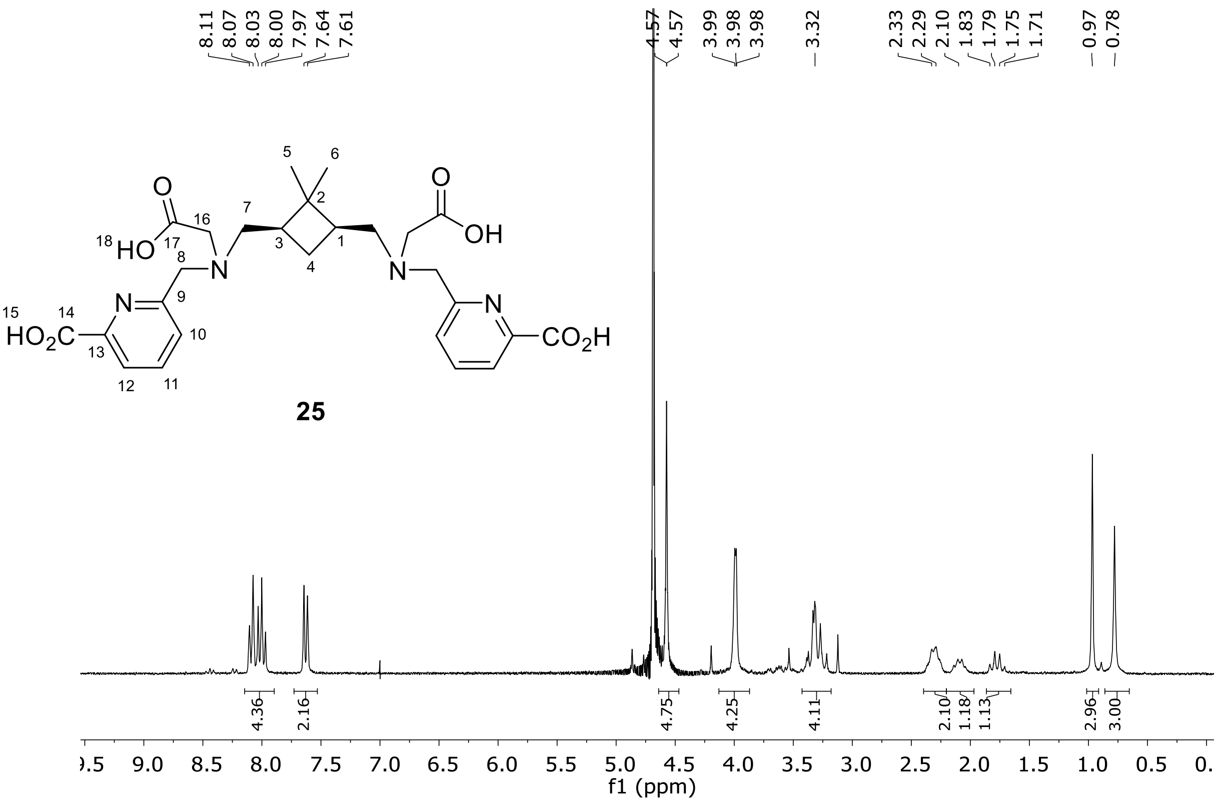

^{13}C-NMR (62.5 MHz, D_2O) of compound **25**

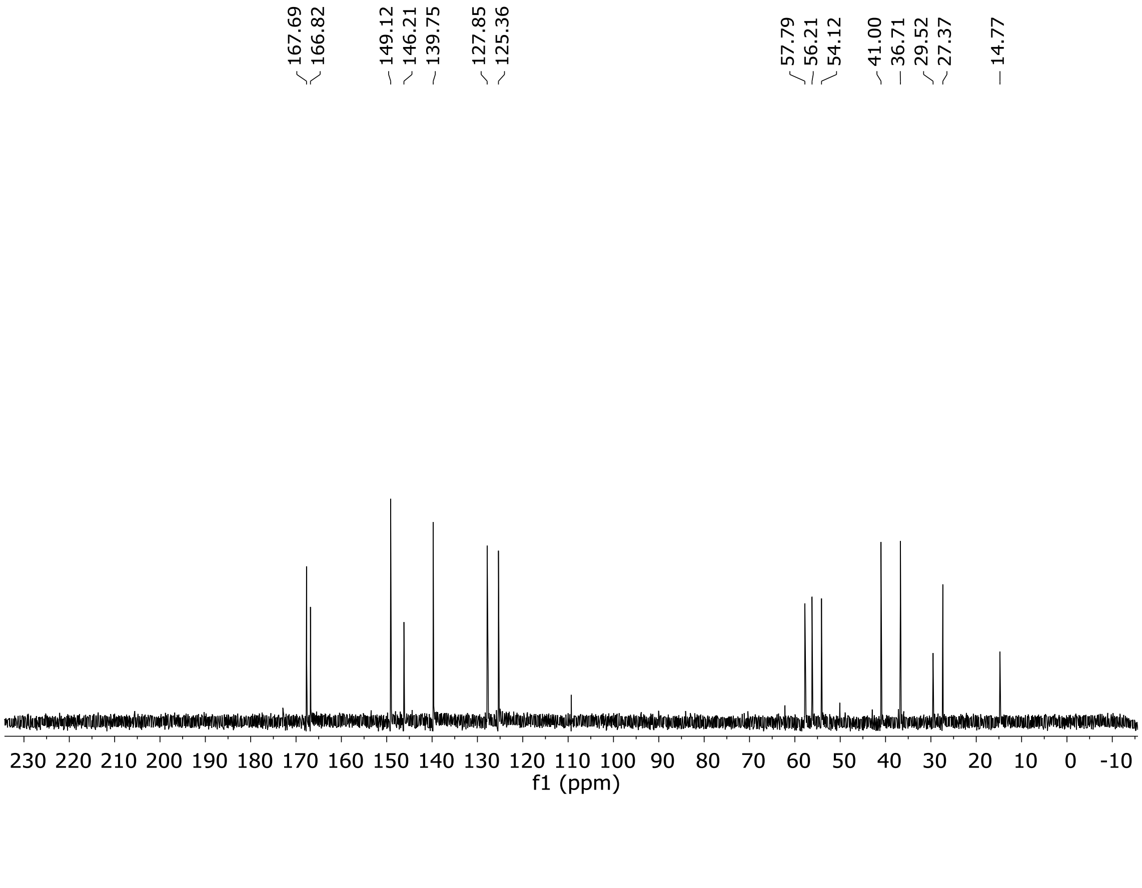

Supplement: Supplementary file 1 [file ijms-20-04333-s001.pdf]
